# Sanitation-related withholding and suppression among women in urban Uganda and India

**DOI:** 10.1038/s44221-025-00452-5

**Published:** 2025-06-17

**Authors:** Elaina Sinclair, Anke Hüls, Madeleine Patrick, Srishty Arun, Vinod Ramanarayanan, Sheela S. Sinharoy, Bethany A. Caruso

**Affiliations:** 1 Department of Epidemiology, Rollins School of Public Health, Emory University, Atlanta, GA USA; 2 Gangarosa Department of Environmental Health, Rollins School of Public Health, Emory University, Atlanta, GA USA; 3 Hubert Department of Global Health, Rollins School of Public Health, Emory University, Atlanta, GA USA; 4 Civic Fulcrum, Chennai, India

**Keywords:** Development studies, Psychology and behaviour, Environmental social sciences

## Abstract

Despite sanitation facility availability, perceived privacy, safety and security, and health status and risks may prevent toilet use, particularly for women. Women may withhold food and water (withholding) or suppress urination and defecation urges (suppression) to cope, though evidence on the prevalence and drivers of these behaviours is limited. This secondary analysis of data generated as part of the Measuring Urban Sanitation and Empowerment project (*n* = 2,173) assesses the prevalence of withholding and suppression among urban women in Kampala, Uganda and Tiruchirappalli, India, and associations with perceived sanitation-related privacy, safety and security, and health status and risks (withholding analytic sample, 1,308; suppression analytic sample, 862). Witholding was reported by 38% (265/697) of women in Kampala and 16% (100/611) in Tiruchirappalli; more than 93% of women in both populations (Kampala, 415/440; Tiruchirappalli, 336/350) reported suppression. Privacy, safety and security, and health scores were all significantly associated with the odds of withholding in both cities. Fewer significant results were found from linear regression analyses assessing privacy, safety and health scores and suppression, suggesting other, unaccounted-for influences. The results suggest that sanitation-related privacy, safety and health conditions should be addressed programmatically to improve women’s sanitation-related circumstances and behaviours.

## Abstract (web summary)

As a result of unsupportive sanitation environments, women may cope by suppressing urges to urinate and defecate or by not eating food and/or drinking water. Among urban women in Uganda and India, nearly all women surveyed reported suppressing urination and defecation urges. Withholding food and water was less common. Perceived privacy, safety and health influenced these coping behaviours.

## Main

Despite the expansion of safe water, sanitation and hygiene access around the world, more than 1.9 billion households are estimated to lack access to at least a basic sanitation facility, meaning one that is designed to hygienically separate excreta from human contact and is not shared with other households^1^. Inadequate progress in increasing coverage compromises the achievement of Sustainable Development Goal 6.2, which calls for universal access to adequate and equitable sanitation, with special attention to the needs of women and girls^2^. Importantly, however, having facility access does not guarantee use. A post hoc regression analysis found that every 10% increase in latrine coverage resulted in only a 5.8% increase in use, demonstrating that behaviours are not driven by access alone^3^. The lack of use among those owning household latrines has even inspired interventions to increase use, with mixed results^4–7^. Findings suggest that people’s perceptions of both physical and social sanitation environments are critical determinants of use, regardless of facility availability. Research has shown that women’s sanitation needs specifically may be compromised by broader social and physical environments, despite access^8–11^. In the face of unsupportive sanitation environments, women may cope by holding or suppressing urges to urinate and defecate (suppression) or by not eating food and/or drinking water (withholding)^12^. To ensure that women’s sanitation needs are met, there is a need to further understand the extent of suppression and withholding behaviours and what influences them^13^.

Women have reported myriad physical and social environmental factors that influence their sanitation behaviours, including perceived cleanliness, privacy and health risks at available sanitation locations; facility use fees; fear of gender-based violence at or on the way to sanitation locations; social expectations such as prioritizing family responsibilities (for example, cooking) over their own needs; requiring accompaniment to sanitation locations for safety and dignity reasons; menstrual health and hygiene needs, attending to needs only at specific times (for example, dawn or dusk) to prevent shame; and inadequate facility design features or available resources within to meet their or their dependents’ needs (for example, lacking disposal bins for menstrual materials or diapers/nappies)^9,10,14–24^. Acknowledging these factors and to better meet the physical and health needs of women, there have been calls for ‘female-friendly’ public and community toilets that are safe, private, conveniently and appropriately located, accessible, affordable, gender separated, well managed to ensure cleanliness and resourced to meet the menstruation and caregiving needs of users (for example, water, soap, disposal and so on)^13,25^. In the face of unsupportive conditions and circumstances, women may develop coping strategies that compromise their ability to meet their needs and potentially jeopardize their overall well-being. Specifically, by withholding food or water or suppressing urges, women can potentially limit the frequency with which they need to access unsupportive sanitation environments^8,11,12,17,24,26–28^. However, while these behaviours may be a means of coping in the short term, women who have withheld food and water or suppressed urination and defecation have reported frequent urinary tract infections, headaches, stomach aches, constipation, diarrhoea and other illnesses^11,28–30^.

Quantitative research on the prevalence of withholding and suppression behaviours among women is limited. A 2020 study across three cities in Maharashtra, India, assessed whether women ‘ever avoided urination or defecation’ or ‘ever restricted food or liquid to avoid urination or defecation’ before and after construction of a sewer-connected household toilet^12^. The authors found that the proportion of women who ‘ever avoided urination or defecation’ reduced from 16.6% before construction to 2.2% after construction, and those who ‘ever restricted food or liquid’ reduced from 21.5% to 4.9%. They also found perceived risk to safety and lack of water availability to be significantly associated with the assessed avoidance and restriction behaviours^12^. These findings suggest the importance of toilet environments and experience in reducing suppression and withholding behaviours and underscore the need for further learning about these behaviours.

The aims of this research were to describe the prevalence of withholding and suppression among urban women in Kampala, Uganda and Tiruchirappalli, India, and to identify the privacy, safety and security, and health factors associated with sanitation-related withholding and suppression.

## Withholding was less common and suppression was ubiquitous

Among respondents in Kampala, 38.0% reported some level of withholding in the previous 30 days, compared with 16.4% in Tiruchirappalli (Table 1). For both populations, withholding water when away from home (33.3% (*n* = 232) in Kampala and 12.8% (*n* = 78) in Tiruchirappalli) was the most common withholding scenario as compared with withholding food when away from home or withholding food or water at home (Supplementary Table 3). Some level of suppression in the previous 30 days was near universal in both populations (94.3% (*n* = 415) in Kampala and 96.0% (*n* = 336) in Tiruchirappalli). In both populations, the number of women who reported any level of suppression at night (87.5% (*n* = 385) in Kampala and 92.9% (*n* = 325) in Tiruchirappalli) was much greater than those who reported any level of suppression when home during the day (20.2% (*n* = 89) in Kampala and 8.9% (*n* = 31) in Tiruchirappalli) or when away from home (40.0% (*n* = 176) in Kampala and 14.0% (*n* = 49) in Tiruchirappalli) (Supplementary Table 4). The mean suppression scores were near 2, indicating a mean suppression frequency of ‘sometimes’ (Kampala 1.97, s.d. of 0.47 and Tiruchirappalli 1.91, s.d. of 0.38).

**Table 1:** Descriptive statistics of outcomes and primary exposures for participants in Kampala and Tiruchirappalli

	Withholding dataset		Suppression dataset	
	Kampala (*n* = 697)	Tiruchirappalli (*n* = 611)	Kampala (*n* = 440)	Tiruchirappalli (*n* = 350)
Withholding^a^, % (*n*)	38.00 (265)	16.40 (100)	Not applicable	Not applicable
Suppression^a^, % (*n*)	Not applicable	Not applicable	94.30 (415)	96.00 (336)
Suppression score, mean (s.d.)	Not applicable	Not applicable	2.00 (0.47)	1.90 (0.38)
Privacy scale, mean (s.d.)	1.19 (0.51)	0.90 (0.29)	1.22 (0.53)	0.93 (0.32)
Safety and security scale, mean (s.d.)	1.86 (0.54)	1.68 (0.43)	1.88 (0.55)	1.71 (0.46)
Safety and security factor 1, mean (s.d.)				
Perceptions of women’s general risk of harm when going for sanitation	1.93 (0.73)	1.77 (0.67)	1.94 (0.76)	1.80 (0.71)
Safety and security factor 2, mean (s.d.)				
Perceptions of women’s risk of harm when going to sanitation-related meetings	1.81 (0.68)	1.76 (0.67)	1.79 (0.70)	1.78 (0.69)
Safety and security factor 3, mean (s.d.)				
Perceptions of women’s risk of domestic violence related to sanitation	2.01 (0.73)	2.01 (0.71)	2.02 (0.74)	2.01 (0.70)
Safety and security factor 4, mean (s.d.)				
Perceptions of one’s own risk of harm when going for sanitation	1.53 (0.66)	1.21 (0.52)	1.56 (0.66)	1.27 (0.60)
Safety and security factor 5, mean (s.d.)				
Perceptions of general personal safety related to sanitation	1.66 (0.74)	1.34 (0.59)	1.71 (0.74)	1.36 (0.60)
Health factor 1, mean (s.d.)				
Sanitation-related illness	1.62 (0.65)	1.05 (0.22)	1.66 (0.66)	1.07 (0.25)
Health factor 3, mean (s.d.)				
Fear of injury	1.26 (0.55)	1.07 (0.28)	1.26 (0.56)	1.09 (0.32)
Health factor 4, mean (s.d.)				
Sanitation-related anxiety, embarrassment and shame	1.25 (0.53)	1.06 (0.28)	1.26 (0.53)	1.08 (0.33)
Health factor 5, mean (s.d.)				
Sanitation-related stress and fear	1.75 (0.78)	1.16 (0.40)	1.81 (0.79)	1.19 (0.47)

^a^Includes all women who reported performing the noted behavior—whether sometimes, often, or always—on one or more related survey items.

## Sanitation sharing more common among the Kampala sample

In Kampala, all women (100.0%) used improved sanitation facilities (those designed to hygienically separate excreta from human contact; specifically flush toilet, pit latrine or composting toilet) for defecation and most shared their sanitation facility with known households (75.6%). In Tiruchirappalli, most women used improved sanitation facilities (77.7%) and had privately owned sanitation facilities (69.2%). Most women in Kampala (91.7%) had to collect water for household sanitation needs, compared with 31.1% of women in Tiruchirappalli.

Additionally, the sample population in Kampala was younger than in Tiruchirappalli, but this difference was smaller between suppression datasets (mean age: Kampala 30.3 years and Tiruchirappalli 32.1 years) than withholding datasets (mean age: Kampala 32.2 years and Tiruchirappalli 40.9 years) (Table 2).

**Table 2:** Descriptive statistics of covariates for sampled women in Kampala and Tiruchirappalli

	Withholding		Suppression	
	Kampala (*n* = 697)	Tiruchirappalli (*n* = 611)	Kampala (*n* = 440)	Tiruchirappalli (*n* = 350)
Age, mean (s.d.)	32.2 (10.7)	40.9 (14.9)	30.3 (8.0)	32.1 (8.2)
Wealth index^a^, mean (s.d.)	2.8 (1.4)	2.9 (1.4)	2.7 (1.3)	3.0 (1.4)
Number of household members, mean (s.d.)	4.4 (2.2)	4.3 (3.3)	4.3 (2.1)	4.5 (2.7)
Number of household children, mean (s.d.)	0.8 (0.4)	0.6 (0.5)	0.8 (0.4)	0.7 (0.4)
Hours away from home, mean (s.d.)	4.7 (4.4)	3.0 (3.3)	4.9 (4.6)	3.0 (3.2)
Self-rated physical health, % (*n*)				
Excellent	6.9 (48)	10.0 (61)	7.3 (32)	12.9 (45)
Very good	21.2 (148)	12.8 (78)	22.5 (99)	15.1 (53)
Good	48.3 (337)	36.5 (223)	49.1 (216)	42.00 (147)
Fair	15.1 (105)	35.3 (216)	13.6 (60)	27.7 (97)
Poor	8.5 (59)	5.4 (33)	7.5 (33)	2.3 (8)
Marital status, % (*n*)				
Never married	16.8 (117)	10.1 (62)	22.3 (98)	16.7 (45)
Married	47.5 (331)	75.0 (458)	43.4 (191)	80.7 (218)
Separated/divorced/widowed	35.7 (249)	14.9 (102)	34.5 (151)	2.6 (7)
Facility type % (*n*)				
Improved	100 (697)	77.7 (475)	100 (440)	77.1 (270)
Unimproved or open defecation^b^	0 (0)	22.3 (136)	0 (0)	22.9 (80)
Facility location^c^, % (*n*)				
In own dwelling	6.7 (47)	63.7 (389)	6.4 (28)	63.7 (223)
In own yard/plot	79.1 (551)	15.7 (96)	78.6 (346)	16.0 (56)
Elsewhere	14.2 (99)	20.6 (126)	15.0 (66)	20.3 (71)
Share facility, % (*n*)				
Privately owned/not shared	13.2 (92)	69.2 (423)	10.9 (48)	67.4 (236)
Known households	75.6 (527)	10.1 (62)	77.9 (343)	12.9 (45)
General public	11.2 (78)	20.6 (126)	11.1 (49)	19.7 (69)
Private location, % (*n*)	83.6 (583)	37.8 (231)	82.9 (365)	35.1 (123)
Could be seen using, % (*n*)	16.9 (118)	4.7 (29)	17.3 (76)	4.9 (17)
Men also use the facility, % (*n*)	96.8 (675)	77.4 (473)	96.4 (424)	80.6 (282)
Lockable facility, % (*n*)	82.8 (577)	72.2 (442)	81.1 (357)	73.1 (256)
Lighting inside facility, % (*n*)	64.4 (449)	74.6 (456)	67.0 (295)	74.0 (259)
Lighting outside/on the way, % (*n*)	79.3 (553)	96.1 (587)	70.3 (349)	96.6 (338)
Physically challenging to access, % (*n*)	13.6 (95)	10.0 (61)	14.3 (63)	7.1 (25)
Facility malfunction, % (*n*)	17.9 (125)	6.9 (42)	17.9 (79)	7.7 (27)
Collect water for household sanitation needs, % (*n*)	91.7 (639)	31.1 (190)	92.0 (405)	34.3 (120)

^a^Wealth index values were calculated by country using the World Health Organization’s International Wealth Index.

^b^Fewer than ten participants reported open defecation.

^c^Facility location was colinear with share facility and was not used as a covariate.

## Women have privacy, safety and health concerns

Privacy and safety and security scale scores were lower in Tiruchirappalli than in Kampala, indicating that women in Tiruchirappalli had fewer negative experiences and perceptions regarding sanitation-related privacy and safety and security. Both scale scores also were slightly higher in the withholding datasets than the suppression datasets (Table 1). Higher health factor scores and variances were observed among women in Kampala, indicating that women in Kampala had more negative experiences and perceptions regarding sanitation-related health.

## Privacy, safety and health associated with withholding

**Fig. 1:**
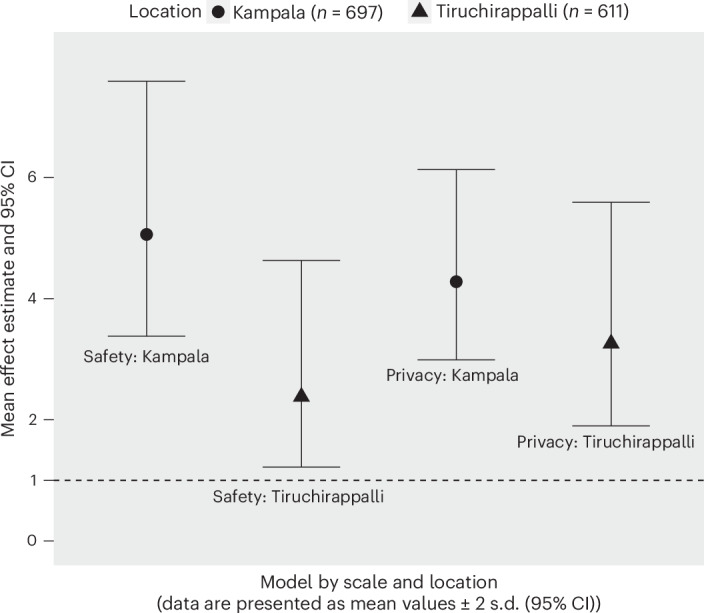
Withholding odds ratios for scale scores. The predicted odds ratios and 95% CIs for the effect of a one-unit increase for the safety and security and privacy scale scores on the odds of withholding for women in Kampala (circles) and Tiruchirappalli (triangles). The horizontal dashed line (*y* = 1) indicates the null value for an odds ratio. A measure of association is not statistically significant if the 95% confidence interval contains the null value.

In both Kampala and Tiruchirappalli, women who reported less sanitation-related privacy, as indicated by higher privacy scale scores, had greater odds of withholding (Table 3 and Fig. 1). For each one-unit increase in the privacy scale score (indicating a lower ability to maintain sanitation-related privacy), the odds of withholding were 5.06 times greater among women in Kampala (95% confidence interval (CI) 3.38 to 7.59) and 2.37 times greater among women in Tiruchirappalli (95% CI 1.22 to 4.63). Privacy scale model outputs for withholding among both populations are available in Supplementary Table 5.

**Table 3:** Logistic regression estimates of the effect of a one-unit increase in scale/factor values on the odds that a woman ever withholds food or water

Scale/factor score	Kampala (*n* = 697)		Tiruchirappalli (*n* = 611)	
	Odds ratio	95% CI	Odds ratio	95% CI
Privacy scale	5.06	3.38 to 7.59	2.38	1.22 to 4.63
Safety and security scale	4.28	2.99 to 6.13	3.26	1.90 to 5.59
Safety and security factor 1: perceptions of women’s risk of harm when going for sanitation	0.89	0.61 to 1.29	1.29	0.78 to 2.14
Safety and security factor 2: perceptions of women’s risk of harm when going to sanitation-related meetings	1.33	0.90 to 1.95	1.27	0.76 to 2.14
Safety and security factor 3: perceptions of women’s risk of domestic violence related to sanitation	1.41	1.04 to 1.90	1.13	0.75 to 1.71
Safety and security factor 4: perceptions of own risk of harm when going for sanitation	3.01	2.11 to 4.29	2.44	1.58 to 3.77
Safety and security factor 5: perceptions of general personal safety related to sanitation	0.90	0.67 to 1.19	0.69	0.43 to 1.10
Health factor 1: sanitation-related illness	1.70	1.25 to 2.31	2.52	1.01 to 6.28
Health factor 3: fear of injury	1.92	1.23 to 2.98	0.85	0.30 to 2.39
Health factor 4: sanitation-related anxiety, embarrassment and shame	1.32	0.80 to 2.20	0.70	0.23 to 2.17
Health factor 5: sanitation-related stress and fear	1.44	1.09 to 1.89	2.93	1.59 to 5.41

Models adjusted for all 18 covariates are listed in Table 2.

Women in Kampala and Tiruchirappalli who reported greater concern for their safety and the safety of other women, as indicated by greater safety and security scale scores, had greater odds of withholding (Table 3 and Fig. 1). For each one-unit increase in the safety and security scale score (indicating more perceived risks), the odds of withholding were 4.28 times greater among women in Kampala (95% CI 2.99 to 6.13) and 3.26 times greater among women in Tiruchirappalli (95% CI 1.90 to 5.59).

In models assessing safety and security factor scores, safety and security factor 3 (perceptions of women’s general risk of domestic violence related to sanitation) and safety and security factor 4 (perceptions of one’s own risk of harm when going for sanitation) were significantly associated with withholding for the Kampala population, and safety and security factor 4 was statistically significant among the Tiruchirappalli population (Table 3 and Fig. 1). Among women in both Kampala and Tiruchirappalli, the strongest association for withholding was found for safety and security factor 4 (perceptions of one’s own risk of harm when going for sanitation); for each one-unit increase in safety and security factor 4, the odds of withholding increased 3.01 times among women in Kampala (95% CI 2.11 to 4.29) and 2.44 times among women in Tiruchirappalli (95% CI 1.58 to 3.77). Among women in Kampala, for each one-unit increase in safety and security factor 3 (perceptions of women’s general risk of domestic violence related to sanitation), the odds of withholding increased 1.41 times (95% CI 1.04 to 1.90). Safety and security scale model outputs for withholding among both populations are available in Supplementary Table 6. Safety and security factor model outputs are available in Supplementary Table 7.

**Fig. 2:**
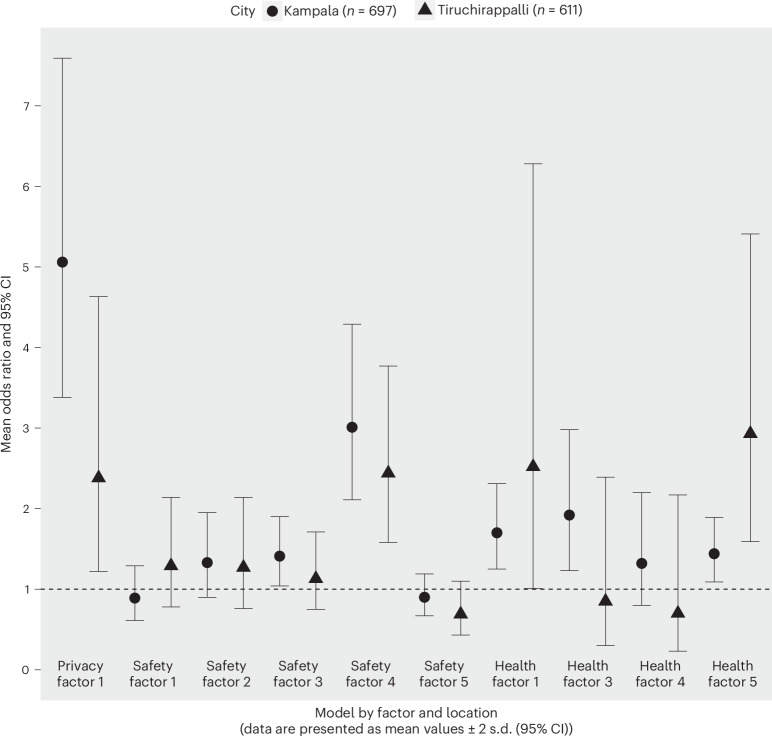
Withholding odds ratios for factor scores. Predicted odds ratios and 95% CIs for the effect of a one-unit increase for a given factor score on the odds of withholding for women in Kampala (circles) and Tiruchirappalli (triangles). The privacy scale has only one factor and is shown again here. The horizontal dashed line (*y* = 1) indicates the null value for an odds ratio. A measure of association is not statistically significant if the 95% confidence interval contains the null value.

Across both populations, women who reported lower levels of sanitation-related health, as indicated by greater health factor scores, had greater odds of withholding (Table 3 and Fig. 2). Three of four health factors assessed were statistically significant for the Kampala population, compared with only two of the four factors for the Tiruchirappalli population. Health factor 4 (sanitation-related anxiety, embarrassment and shame) was not statistically significant in either population (Table 3 and Fig. 2). Among women in Kampala, the strongest association was for health factor 3 (fear of injury). For each one-unit increase in health factor 3 (indicating greater fear of injury), the odds of withholding were 1.92 times greater (95% CI 1.23 to 2.98). Additionally, among women in Kampala, for each one-unit increase in health factor 1 (sanitation-related illness) the odds of withholding were 1.70 times greater (95% CI 1.09 to 1.89), and for each one-unit increase in health factor 5 (sanitation-related stress and fear), the odds of withholding were 1.44 times greater (95% CI 1.09 to 1.89). Among women in Tiruchirappalli, the strongest association was for health factor 5 (sanitation-related stress and fear). For each one-unit increase in health factor 5 (indicating more sanitation-related stress and fear), the odds of withholding were 2.93 times greater among women in Tiruchirappalli (95% CI 1.59 to 5.41). In addition, for each one-unit increase in health factor 1 (sanitation-related illness), the odds of withholding among women in Tiruchirappalli increased by 2.52 times (95% CI 1.01 to 6.28). Health factor model outputs for both populations are available in Supplementary Table 8.

## Mixed associations for suppression

The privacy scale score produced significant model outputs for suppression among women in Tiruchirappalli but not among women in Kampala (Table 4). Specifically, among women in Tiruchirappalli, for every one-unit increase in the privacy scale score (indicative of women feeling they have less privacy), a woman’s suppression score was estimated to increase by 0.20 (95% CI 0.08 to 0.32). Privacy scale model outputs for suppression among women in Kamapala and Tiruchirappalli are available in Supplementary Table 9.

**Table 4:** Linear regression estimates of the effect of a one-unit increase in scale/factor values on the effect estimate of the frequency of suppression

Scale/factor score	Kampala (*n* = 440)		Tiruchirappalli (*n* = 350)	
	Effect estimate	95% CI	Effect estimate	95% CI
Privacy scale	0.03	−0.06 to 0.12	0.20	0.08 to 0.32
Safety and security scale	0.02	−0.06 to 0.11	0.09	0.00 to 0.18
Safety and security factor 1: perceptions of women’s risk of harm when going for sanitation	−0.19	−0.29 to −0.10	0.00	−0.09 to 0.09
Safety and security factor 2: perceptions of women’s risk of harm when going to sanitation-related meetings	0.13	0.03 to 0.22	0.01	−0.08 to 0.10
Safety and security factor 3: perceptions of women’s risk of domestic violence related to sanitation	0.07	−0.01 to 0.14	0.00	−0.07 to 0.07
Safety and security factor 4: perceptions of own risk of harm when going for sanitation	0.04	−0.04 to 0.13	0.13	0.06 to 0.21
Safety and security factor 5: perceptions of general personal safety related to sanitation	−0.02	−0.09 to 0.04	−0.06	−0.13 to 0.01
Health factor 1: sanitation-related illness	0.07	−0.01 to 0.15	−0.02	−0.20 to 0.16
Health factor 3: fear of injury	0.07	−0.04 to 0.18	0.20	−0.01 to 0.42
Health factor 4: sanitation-related anxiety, embarrassment and shame	−0.15	−0.28 to −0.02	−0.12	−0.33 to 0.09
Health factor 5: sanitation-related stress and fear	0.01	−0.06 to 0.08	0.04	−0.08 to 0.15

Models adjusted for all 18 covariates are listed in Table 2.

Similarly, the overall safety and security scale score produced significant model outputs for suppression among women in Tiruchirappalli but not among women in Kampala (Table 4). Specifically, among women in Tiruchirappalli, for every one-unit increase in the safety and security scale score (indicative of women feeling less safe and less secure), a woman’s suppression score was estimated to increase by 0.09 (95% CI 0.00 to 0.18). Safety and security scale model outputs for suppression among women in Kamapala and Tiruchirappalli are available in Supplementary Table 10.

Varied safety and security factor scores were found to be associated with suppression in both Kampala and in Tiruchirappalli; safety and security factors 1 (perceptions of women’s general risk of harm when going for sanitation) and 2 (perceptions of women’s risk of harm when going to sanitation-related meetings) were statistically significant for the Kampala population, while only safety and security factor 4 (perceptions of one’s own risk of harm when going for sanitation) was significant for the Tiruchirappalli population (Table 4 and Fig. 2). Specifically, among women in Kampala, for each one-unit increase in safety and security factor 2 (perceptions of women’s risk of harm when going to sanitation-related meetings), their suppression score was estimated to increase by 0.13 (95% CI 0.03 to 0.22). An inverse relationship was observed for safety and security factor 1 (perceptions of women’s general risk of harm when going for sanitation), where a one-unit increase was associated with a decrease in a woman’s suppression of 0.19 (95% CI −0.29 to −0.10) indicative of less suppression. Among women in Tiruchirappalli, for each one-unit increase in safety and security factor 4 (perceptions of one’s own risk of harm when going for sanitation), their suppression score was estimated to increase by 0.13 (95% CI 0.06 to 0.21). Safety and security factor model outputs for suppression are available in Supplementary Table 11.

No health factors had statistically significant associations among women in Tiruchirappalli and only one did in Kampala (factor 4). Among women in Kampala, for each one-unit increase in health factor 4 (sanitation-related anxiety, embarrassment and shame), their suppression score was estimated to decrease by 0.14 (95% CI −0.28 to −0.02). Health factor model outputs for suppression are available in Supplementary Table 12.

## Discussion

The aims of this study were to describe the prevalence and frequency of sanitation-related withholding and suppression among urban women in Kampala and Tiruchirappalli, and to identify if and how privacy, safety and security, and health were associated with these often-overlooked sanitation coping behaviours. Withholding was a coping behaviour employed by women of both populations, but more so in Kampala (38.0%) than Tiruchirappalli (16.4%). Suppression was nearly ubiquitous with 94.3% of women in Kampala and 91.1% of women in Tiruchirappalli reporting some level of suppression. Predictors vary by behaviour and population.

### Predictors of withholding and suppression can be modified

Analysis found higher privacy, safety and security, and health scores (indicating more negative experience) to be significant predictors of withholding. Fewer significant results were attained for suppression, probably due to it being a commonly reported behaviour. Nonetheless, sanitation-related privacy and the perception of risk of harm when going for sanitation are associated with suppression for women in Tiruchirappalli. Privacy, safety and security, and health are modifiable, and thus should be addressed to reduce women’s needs to practice these potentially harmful coping behaviours.

While the mean suppression score in both cities implies that suppression was infrequent (between never and sometimes in the past 30 days), the long-term clinical consequences of both withholding and suppression, such as urinary tract infections, headaches, stomach aches, constipation and other illnesses, may be of concern for the 1.7% and 6.2% of all surveyed women who reported ‘often’ or ‘always’ engaging in withholding or suppression behaviours, respectively^11,27,30^. Women practising these coping behaviours with any frequency have had to compromise their bodily needs and therefore live in physical and/or social environments that are not wholly supportive. As reported by numerous other studies, sanitation coping strategies can cause stress and adversely affect mental well-being^8,18,19,31,32^. More research is needed to understand how these behaviours are associated with these and other health outcomes.

### Withholding and suppression research is limited

Studies assessing withholding and suppression as primary outcomes are limited. Across three major cities in India, Panchang et al. observed that 16.6% of respondents engaged in what they called general avoidance (analogous to suppression) and 21.5% reported diet restriction (analogous to withholding)^12^. After sanitation improvements, Panchang et al. found reductions in these behaviours, with 2.2% of women reporting general avoidance and 4.9% reporting diet restriction^12^. In the current study, the prevalence of suppression in both locations and withholding in Kampala (38.0%) were all much higher than seen in Panchang et al., though the prevalence of suppression in Tiruchirappalli (16.4%) was similar^12^. In Uganda, Borg et al. report that 60.9% of female respondents working in Mukono District marketplaces needed to sometimes or always delay urinating (suppression) while working in the past month^29^. This is a smaller percentage of women who engage in suppression than is reported by the present study; however, the present study does not differentiate between suppressing the urge to urinate or defecate and includes women who rarely do so.

### Perceived safety influences withholding and suppression

Consistent with existing literature, perceptions of one’s own risk of harm when going for sanitation (safety and security factor 4) had the strongest association with both withholding among women in both populations and suppression in Tiruchirappalli^11,17,18,20,22,23^. The strength of this association is supported by social norms in Tamil Nadu, which discourage women from using public sanitation facilities alone due to beliefs it is neither safe nor proper to do so; the threat of violence to women when accessing sanitation, especially from non-family members, is especially prevalent among poor urban women^33^. The findings in the present study also align with those of Panchang et al.^12^, who found that Indian women who reported their local community toilet block to be unsafe had over four times the odds of reporting avoidance (suppression) and over three times the odds of reporting restriction (withholding) at baseline compared with those reporting their sanitation facility to be safe. In this study, the odds ratios for associations between safety and security factor 4 (perceptions of one’s own risk of harm when going for sanitation) and withholding were higher among women in Kampala than women in Tiruchirappalli, but both were significant. One possible explanation for this difference in the strength of associations could be the greater percentage of women who share facilities with men in Kampala (96.8%) compared with in Tiruchirappalli (77.4%). Additionally, more women in Kampala reported being able to be seen while using the facility and were less likely to have lockable facilities or facilities with lighting on the way to or inside the facility.

### Influences of privacy and health varied by population

Privacy influences withholding and suppression differently in our study populations, and further work is needed to understand these differences. Specifically, while privacy scale scores were significantly associated with the odds of withholding in both populations, with a greater association among women in Kampala, they were only significantly associated with suppression among women in Tiruchirappalli. This incongruence could indicate that women in Kampala proactively cope with a lack of privacy by withholding food or water, whereas women in Tiruchirappalli may be using both coping strategies. These findings are somewhat consistent with the limited body of existing research on sanitation coping behaviours among women. Research from Kampala reported alternative means to relieve urges, such as buckets, plastic bags and even child potties, when sanitation environments were considered unsuitable^34,35^. Suppression is more discussed as a coping strategy in India, where women have described suppressing while open defecating by ‘standing up’ to avoid being seen by approaching people^11,17,36^.

Perceptions related to health were also associated with women’s withholding and suppression behaviours. Among the health factors, ‘fear of injury’ (health factor 3) was associated with the highest odds of withholding in Kampala, and a statistically significant association between ‘sanitation-related stress and fear’ (health factor 5) and withholding was observed in both populations. The fear of injury is closely related to the perception of the risk of harm, and the fear of actual or threatened violence negatively impacts women’s quality of life^17,18,21,23^. Additionally, statistically significant associations were observed between withholding and health factor 1 (sanitation-related illness) in both populations; however, the association was greater among women in Tiruchirappalli (odds ratio of 1.70; 95% CI 1.25 to 2.31) than in Kampala (odds ratio 2.52, 95% CI 1.01 to 6.28).

### Further investigation on suppression needed

More significant results were found for the odds of withholding than the effect estimate for suppression frequency. As noted above, this could be because suppression was common among study respondents in both cities, suggesting that a more nuanced measure of suppression may be needed. For example, in addition to frequency, it may be worthwhile to measure average duration of suppression, as suppression for longer periods of time may have more serious health consequences. The difference in modelling success between the two coping strategies also indicates unmeasured variables that could be influencing women’s suppression. Further investigation is needed to better understand the causal factors and motivating circumstances under which women suppress.

### Limitations

This study has several limitations. Women were asked about withholding and suppression events occurring in the previous 30 days and their responses may been affected by recall bias. Future research could investigate the frequency and/or duration of withholding and suppression within the past week. Additionally, future research could conduct surveys at varying times of the year to assess any potential impact of seasonal variability on outcomes.

As this is a secondary analysis with data collected without this analysis in mind, other influencing exposures or covariates could be considered in future research that were not included here, including the availability of paper, water and soap for personal hygiene and facility cleanliness. Relatedly, the survey did not include questions that enabled precise classification of sanitation according to the JMP ladder. Sanitation facilities were classified based on improved or unimproved status based on type; hanging, bucket and flying latrines were considered unimproved and other latrine types were considered improved, which may be imprecise. Few participants reported open defecation and were grouped with unimproved facilities. No information was available on faecal sludge management. Future work could include these variables. Further, this study only investigated the perceptions and experiences of adult women. Future research could broaden the population investigated to understand whether, how, and why experiences may vary by age, gender, disability status and so on.

There were missing data issues, most notably regarding one of the suppression items (suppression at night), which led to additional data restriction and fewer observations among the suppression datasets. It is not certain whether the data are missing completely at random; linear and logistic regression were completed using complete cases without data imputation. Last, by using scale and factor scores as the exposure of interest, each domain (privacy, safety and security, and health) is limited to only their component survey items. The scale and factor scores were not developed specifically to predict withholding and suppression, and therefore there may be additional privacy, safety and security, or health-specific survey items that were not analysed, but may influence women’s use of withholding or suppression.

Nonetheless, this study demonstrated the utility of analysis by health factors rather than by the overall health scale score and demonstrated the ability to explore safety and security by both scale and factors. Key strengths of this analysis were the large samples of urban women in both Kampala and Tiruchirappalli and the breadth of data collected on covariates. Additionally, this is one of the few quantitative studies able to analyse withholding and suppression as primary outcomes, and the only one known to do so in more than one country.

## Conclusions

This study documented levels of withholding and suppression among survey respondents in Kampala and Tiruchirappalli and identified associated privacy, safety and security, and health factors. Further refinement of the model and exploration of other Agency, Resources, Institutional Structures for Empowerment (ARISE) scale domains should be pursued to more completely identify other sociocultural and physical factors that may be associated with withholding and suppression behaviours. The influencing factors identified in this study, particularly perceived risk of harm, fear of injury and sanitation-related stress and fear, can inform programmatic decisions and help target sanitation interventions to improve women’s sanitation experiences and reduce the need for women feel the need to use coping mechanisms such as withholding and suppression.

## Methods

### Ethics

Study activities were reviewed and approved by institutional review boards at Emory University, USA (IRB no. 00110271), Azim Premji University, India (reference no. 2019/SOD/Faculty/5.1) and Makerere University, Uganda (reference no. 2019–038). All participants provided oral or written consent to enumerators in their local language using a standardized script. Participants in Uganda received UGX 10,000 (∼US$2.71) in accordance with local policies and ethical requirements. A total of 2,173 women over the age of 18 years participated in the survey for the parent study: 1,094 in Kampala and 1,080 in Tiruchirappalli. All enumerators were local women fluent in the local language(s). Consent was obtained for the primary data collection and any subsequent secondary analyses. All research activities pertaining to this study were performed in accordance with the Declaration of Helsinki.

### Agency, resources and institutional structures for empowerment

This is a secondary analysis of data from the Measuring Urban Sanitation and Empowerment (MUSE) project, which aimed to develop and validate quantitative scales to measure women’s sanitation-related empowerment in urban areas of low-and middle-income countries (see the protocol^37^). For scale development, the survey included items representing three domains (resources, agency and institutional structures) and multiple subdomains of empowerment, adapted from an existing conceptual model for women’s and girls’ empowerment^38^. Factor analysis item response theory and reliability and validity testing were used to create and validate the scales, called the ARISE scales^37,39^. The survey also collected information on participant demographics and water and sanitation conditions. For details on how the ARISE scales were developed, validated and can be used, please see the protocol paper, methodology papers and user guide^37,39–41^.

### Data collection

Cross-sectional data were collected in two locations: Kampala, Uganda, and Tiruchirappalli, India, from December 2019 to January 2020. In brief, Kampala and Tiruchirappalli were both purposively selected from cities participating in the Citywide Inclusive Sanitation (CWIS) programme funded by the Bill and Melinda Gates Foundation. Local CWIS implementing partners and government officials worked with the MUSE research team to purposively select 23 neighbourhoods in Tiruchirappalli and 10 parishes in Kampala, with a focus on low- to middle-income neighbourhoods. In each city, random sampling procedures were used to select households within each neighbourhood or parish, specifically seeking an adult woman within each selected household^39^.

Data were collected during the dry season (average temperature, 64.4–87.1 °F; average precipitation, 0.09 inches (ref. ^42^)) in Kamapala, Uganda’s largest city. Kampala is home to an estimated 1.5 million residents and sees a doubling in population during the day due to transient populations and commuting workers^43^. According to a 2017 Kampala Capital City Authority study, the three major types of sanitation facilities in Kampala are unlined pit latrines (38%), lined pit latrines (30%) and flush toilets (33%)^43^. Approximately 58% of the Kamapala population has access to an improved sanitation facility, which is a facility designed to keep excreta hygienically separated from human contact^44^. Kampala Capital City Authority reported that approximately 50% of all sanitation facilities are shared between an average of five households, or 28 people^43^. Research on sanitation perceptions and behaviours in Kampala is limited. A 2010 cross-sectional study with 1,500 residents found sharing facilities and unhygienic conditions to be top reasons for facility dissatisfaction, while lockability, lighting, dwelling proximity, and day and night accessibility to be reasons for satisfaction; however, assessment of how these perceptions drove behaviour was not conducted and data were not disaggregated to determine gender differences^45^. Qualitative studies identified concerns about being seen or heard (privacy), experiencing injury or harassment on the way to or at the facility (safety), and contracting diseases due to unhygienic conditions (health) to be of greater concern for woman than men, and influenced whether women used facilities or chose buckets, bags, child potties or going in the open instead^34,35^. Data were also collected during the dry season (average temperature, 65.5–93.6 °F; average precipitation, 0.07 inches^42^) in Tiruchirappalli, the fourth largest city in the Indian state of Tamil Nadu with a population near 850,000 (ref. ^46^). In 2018, the Tamil Nadu Urban Sanitation Support Programme published a water and sanitation census for Tiruchirappalli, reporting that 81% of households have access to private toilets, 14% use community or public toilets and 5% practice open defecation^46^. Additionally, 45% of households are connected to the city sewer, 28% are connected to septic tanks, 3% have pit latrines and the remaining 5% of households dispose waste in the open^46^. There is limited research on sanitation behaviours in Tamil Nadu compared with India more broadly. Research in India has found inadequate privacy; fear of being shamed, harmed by animals or men, scared by ghosts or reprimanded by family members; and perceived risks of catching diseases to influence women to hold or suppress urination and defecation needs, and to limit food and water to aid in that suppression^11,17,27,36^. In Tamil Nadu, a 2020 cross-sectional study with 2,427 urban participants found women perceived a greater risk of social sanctions related to open defecation compared with men. While the study did not discuss suppression or withholding food or water, it is plausible that women practice these coping behaviours in Tamil Nadu to avoid social sanctions, particularly if they lack access to a latrine^47^.

### Inclusion criteria and eligibility

To be eligible for participation in the survey (parent study), a woman needed to be 18 years or older, speak Luganda (in Uganda) or Tamil (in India) or English, be able understand the survey and consent, and have no speech or hearing impediments (to avoid comprehension difficulties). Data were collected in each city by enumerator teams comprised of women fluent in the local language using Android tablets equipped with ODK Collect. Team members participated in a 5 day training, which covered the survey, research ethics and logistics, and pilot tested the survey. Data collection was supervised by at least one city coordinator and/or field supervisor per city. Raw data were uploaded on a rolling basis to Emory OneDrive. A complete dataset was uploaded at the conclusion of data collection in each location. A total of 2,173 women participated in the survey for the parent study: 1,094 in Kampala and 1,080 in Tiruchirappalli^39^. Among those, only women who answered all relevant primary outcome, exposure and covariate items were included in this secondary analysis.

### Primary outcomes

Primary outcomes of interest are (1) withholding food and water and (2) suppression of the urge to urinate or defecate, both during the 30 days before the survey.

To assess the frequency of withholding, four survey items were used to create a dichotomous variable of whether a woman ever practised withholding in the previous 30 days. Duration of withholding was not assessed. Withholding items asked whether a woman (1) withheld water at home, (2) withheld water when she knew she would be away from home, (3) withheld food at home and (4) withheld food when she knew she would be away from home. Response options for all withholding items were ‘never’ (coded as 0), ‘sometimes’ (1), ‘often’ (2) or ‘always’ (3). As few women reported any withholding (Supplementary Fig. 1), and those who did reported doing so rarely, the creation of a dichotomized withholding variable was created for this analysis. The dichotomous variable captures whether a woman reported never withholding or reported any level of withholding—whether sometimes, often or always—across the four relevant items (Supplementary Fig. 2).

To assess frequency of suppression, three items regarding suppression were used to create a mean score reflecting the frequency of suppression in the previous 30 days. Duration of suppression was not assessed by the survey. Suppression items asked whether women suppressed the urge to urinate or defecate (1) during the daytime at home, (2) when away from home and (3) at night when at home. Response options for all suppression items were ‘never’ (coded as 0), ‘sometimes’ (1), ‘often’ (2) or ‘always’ (3). Nearly all women reported some level of suppression, enabling the creation of a suppression score. The suppression score, which is an average across all suppression items (for example, the sum of all individual item scores divided by 3), represents the mean frequency with which a woman suppressed, with numbers corresponding with the item response options (never (0) to always (3)) (Supplementary Fig. 3).

### Primary exposures

The primary exposures in this secondary analysis were privacy, safety and security, and health. These exposures were selected based on existing literature in Uganda, India and beyond that describe how perceived risks to privacy, safety and health can influence women’s decision to either use sanitation facilities or avoid them by using alternative locations (for example, buckets, bags or open fields), suppressing urges or withholding food and water^11,17,22,27,28,33–36,48,49^.

Privacy, safety and security, and health were each measured using the corresponding ARISE scales (see Supplementary Table 1a–c for the survey items in each scale)^37,39^. As noted in the protocol and the user guide, the ARISE scales were specifically designed and validated to be administered to adult women living in urban areas^37,39,41^. As such, the scales and the associated survey items specifically consider the gendered experiences of women. For a complete list of scales, factors and survey items, see Supplementary Table 1a–c.

Response options for each scale item (‘never’ (coded value of 0), ‘sometimes’ (1), ‘often’(2) or ‘always’(3)) represent how often a woman experienced, felt or perceived a negative experience or situation related to the scale domain or factor subdomain. Scale item responses were reverse coded so that more frequent negative experiences had higher values. Scale scores are the mean value of all responses to items included in the scale, and factor scores are the mean of all response values from items within the factor. Higher scale or factor scores indicate a greater frequency of negative experiences, feelings or perceptions regarding the scale domain or factor subdomain. Two of the scales (safety and security and health) are multidimensional, meaning they include several subconstructs or factors.

Privacy was defined as ‘women’s ability to maintain desired levels of privacy when accessing and using sanitation locations’^37^. The privacy scale has a single factor, which is composed of five items (Supplementary Table 1a).

Safety and security was defined as “women’s freedom from acts or threats of violence (physical or sexual), coercion, harassment or force when accessing and using sanitation locations or engaging in sanitation-related decision-making processes in the public sphere.”^37^ The safety and security scale includes five factors: (1) ‘perceptions of women’s risk of harm when going for sanitation’, (2) ‘perceptions of women’s risk of harm when going to sanitation-related meetings’, (3) ‘perceptions of women’s risk of domestic violence related to sanitation’, (4) ‘perceptions of participant’s own risk of harm when going for sanitation’ and (5) ‘perceptions of general personal safety related to sanitation’. Each safety and security factor is composed of three or four items (Supplementary Table 1b).

Health was defined as “women’s complete physical, mental and social well-being as affected by sanitation options and conditions; not merely the absence of disease or infirmity.” The health scale includes five factors: (1) ‘sanitation-related illness’, (2) ‘illness due to suppression and withholding’, (3) ‘fear of injury’ (which includes injury by men, boys or other people; animals or insects; and/or physical/environmental conditions when accessing sanitation), (4) ‘sanitation-related anxiety, embarrassment, and shame’ and (5) ‘sanitation-related stress and fear’. Each health factor was composed of three or four items. The full health scale (with all factors) could not be used because of identified overlap with factor 2: ‘illness due to suppression and withholding’. Specifically, because suppression and withholding were the outcomes of interest in this analysis, they could not be included as exposures. Since scales were validated with all factors, factor 2 could not be excluded to create a score. Instead, each of the health factor scores (except factor 2) were used in the analysis. (Supplementary Table 1c).

### Covariates

Informed by research on gender and sanitation, 18 covariates were identified as possible confounders: age, self-reported perceived physical health status, marital status, wealth, hours away from home on an average day, whether the respondent collects water for sanitation purposes, type of sanitation facility used most frequently (improved or unimproved/open defecation), where the sanitation facility was located (in own dwelling, in own yard/plot or elsewhere), with whom a sanitation facility was shared, whether the sanitation facility was in a private location, if a woman could be seen using the sanitation facility, if men also used the sanitation facility, if the sanitation facility was lockable, if there was lighting inside the sanitation facility, if there was lighting outside/on the way to the sanitation facility, if it was physically challenging to access the sanitation facility and if the sanitation facility had malfunctioned in the past 30 days (see Supplementary Figs. 4–6 for directed acyclic graphs)^9,14,16,22,27–29,33–36,48,49^.

Facility location (in own dwelling, in own yard/plot or elsewhere) was determined to be colinear (*r* = 0.75, *P* < 0.001) with facility sharing and was dropped from the analysis. The number of household members and number of household children have been identified in qualitative studies as important covariates and thus was included^11^. Wealth was calculated by constructing an asset index for each country based on the World Health Organization’s International Wealth Index (24 items for Uganda and 28 items for India)^50^. A full list of items included in the asset indices are available in Supplementary Table 2.

### Analysis

For this secondary data analysis, the sample only included those women who provided responses to all relevant primary outcome, exposure and covariate items. Owing to missing data, analytic samples for assessing withholding and suppression are different. The analytic sample for withholding included 697 women from Kampala and 611 women from Tiruchirappalli. The analytic sample for suppression included 440 women from Kampala and 350 women from Tiruchirappalli. All analyses were conducted using R version 4.1.1 (2021-08-10).

Descriptive statistics were produced to understand the prevalence and frequency of withholding and suppression as well as the distribution of the scale and factor exposures and the covariates among the analytic sample of urban women from Kampala and Tiruchirappalli.

As withholding was a dichotomized outcome, logistic regression models were run to estimate the odds ratio for the privacy and safety and security scales, all five safety and security factors, and four of the health factors on any level of withholding. Models for withholding were created for each population of women, in Kampala and Tiruchirappalli, and each scale score (privacy, safety and security, and health). Since the safety and security and health scales each had more than one factor, additional models were created that replaced the overall scale score with the factors. To contextualize the odds ratio estimates, a one-unit increase in each scale or factor score represents the change in the mean response option across relevant items from ‘never’ to ‘sometimes’, ‘sometimes’ to ‘often’ or ‘often’ to ‘always’. Models were adjusted for all 18 covariates previously described.

Linear regression models were run to produce effect estimates of the privacy and safety and security scales, and all five safety and security factors, and four of the health factors on suppression frequency, as indicated by the suppression score. As for withholding, suppression models were run for each population of women in Kampala and Tiruchirappalli, first with each scale overall (privacy, and safety and security) and then with factor scores for those with more than one factor (safety and security, and health). The model outputs report how much more likely a woman is to suppress the urge to urinate or defecate (suppression score) based on a given increase in scale or factor scores, with higher scores indicative of greater stress or negative feelings related to privacy, safety and security, or health.

Models were adjusted for all 18 covariates previously described.

Modelling of both withholding and suppression was completed by scale (privacy, safety and security) and factor (safety and security, health). Analysis of safety and security by factors in addition to the scale allowed for understanding of which factors were driving the associations of the scale variable. See Supplementary Text 1 for withholding and suppression scale and factor models.

### Reporting summary

Further information on research design is available in the Nature Portfolio Reporting Summary linked to this article.

## Supplementary information

Supplementary Information Supplementary Tables 1–12, Figs. 1–8 and Text 1.

Reporting Summary

## Data Availability

All data are publicly available via figshare at 10.6084/m9.figshare.28563911 (ref. ^51^).

## References

[1] *Progress on Household Drinking Water, Sanitation and Hygiene 2000–2022: Special Focus on Gender* (World Health Organization (WHO) and United Nations Children’s Fund (UNICEF), 2023).

[2] *Transforming Our World: the 2030 Agenda for Sustainable Development* (United Nations, 2015).

[3] Garn, J. V. et al. The impact of sanitation interventions on latrine coverage and latrine use: a systematic review and meta-analysis. *Int. J. Hyg. Environ. Health* **220**, 329–340 (2017). 27825597 10.1016/j.ijheh.2016.10.001 PMC5414716

[4] Viswanathan, S. S. R. et al. *Improving Households’ Attitudes and Behaviours to Increase Toilet Use (HABIT) in Bihar, India* 3ie Grantee Final Report (International Initiative for Impact Evaluation (3ie), 2019).

[5] Schmidt, W. P. et al. Cluster-randomised trial to test the effect of a behaviour change intervention on toilet use in rural India: results and methodological considerations. *BMC Public Health* **20**, 1389 (2020). 32917160 10.1186/s12889-020-09501-y PMC7488773

[6] Friedrich, M., Balasundaram, T., Muralidharan, A., Raman, V. R. & Mosler, H. J. Increasing latrine use in rural Karnataka, India using the risks, attitudes, norms, abilities, and self-regulation approach: a cluster-randomized controlled trial. *Sci. Total Environ.* **707**, 135366 (2020). 31877399 10.1016/j.scitotenv.2019.135366

[7] Caruso, B. A. et al. Effect of a low-cost, behaviour-change intervention on latrine use and safe disposal of child faeces in rural Odisha, India: a cluster-randomised controlled trial. *Lancet Planet. Health* **6**, e110–e121 (2022). 35150621 10.1016/S2542-5196(21)00324-7 PMC8850376

[8] Caruso, B. A. et al. The association between women’s sanitation experiences and mental health: a cross-sectional study in rural, Odisha India. *SSM Popul. Health* **5**, 257–266 (2018). 30094321 10.1016/j.ssmph.2018.06.005 PMC6077264

[9] Routray, P., Schmidt, W. P., Boisson, S., Clasen, T. & Jenkins, M. W. Socio-cultural and behavioural factors constraining latrine adoption in rural coastal Odisha: an exploratory qualitative study. *BMC Public Health* **15**, 880 (2015). 26357958 10.1186/s12889-015-2206-3 PMC4566293

[10] Bhatt, N. et al. What motivates open defecation? A qualitative study from a rural setting in Nepal. *PLoS ONE* **14**, e0219246 (2019). 31260506 10.1371/journal.pone.0219246 PMC6602253

[11] Caruso, B. A. et al. Understanding and defining sanitation insecurity: women’s gendered experiences of urination, defecation and menstruation in rural Odisha, India. *BMJ Glob. Health* **2**, e000414 (2017). 29071131 10.1136/bmjgh-2017-000414 PMC5640070

[12] Panchang, S. V., Joshi, P. & Kale, S. Women ‘holding it’ in urban India: toilet avoidance as an under-recognized health outcome of sanitation insecurity. *Glob. Public Health* **17**, 587–600 (2021). 10.1080/17441692.2021.1882527 33573517

[13] Schmitt, M. L., Clatworthy, D., Ogello, T. & Sommer, M. Making the case for a female-friendly toilet. *Water* **10**, 1193 (2018).

[14] Hulland, K. R. et al. Sanitation, stress, and life stage: a systematic data collection study among women in Odisha, India. *PLoS ONE* **10**, e0141883 (2015). 26551866 10.1371/journal.pone.0141883 PMC4638353

[15] Abdul Azeez, E. P., Negi, D. P. & Mishra, A. Women’s experiences of defecating in the open: a qualitative study. *Indian J. Gend. Stud.* **26**, 160–170 (2019).

[16] Caruso, B. A. et al. Water, sanitation, and women’s empowerment: a systematic review and qualitative metasynthesis. *PLoS Water* **1**, e0000026 (2022).

[17] Sahoo, K. C. et al. Sanitation-related psychosocial stress: a grounded theory study of women across the life-course in Odisha, India. *Soc. Sci. Med.* **139**, 80–89 (2015). 26164119 10.1016/j.socscimed.2015.06.031

[18] Bapat, M. & Agarwal, I. Our needs, our priorities; women and men from the slums in Mumbai and Pune talk about their needs for water and sanitation. *Environ. Urban.* **15**, 71–86 (2003).

[19] Rheinländer, T., Gyapong, M., Akpakli, D. E. & Konradsen, F. Secrets, shame and discipline: school girls’ experiences of sanitation and menstrual hygiene management in a peri-urban community in Ghana. *Health Care Women Int.* **40**, 13–32 (2019). 29485336 10.1080/07399332.2018.1444041

[20] Singh, K. K. & Mishra, P. Understanding women’s access to sanitation: a study of the slums in Delhi. *J. Soc. Incl. Stud.* **5**, 200–209 (2019).

[21] Barchi, F. & Winter, S. C. Non-partner violence in sub-Saharan Africa and the built environment: a multicountry analysis of the effects of sanitation, water access, and urban settings. *Violence Against Women* **26**, 1101–1119 (2020). 31230569 10.1177/1077801219853370

[22] Datta, A. & Ahmed, N. Intimate infrastructures: the rubrics of gendered safety and urban violence in Kerala, India. *Geoforum* **110**, 67–76 (2020).

[23] Sommer, M., Ferron, S., Cavill, S. & House, S. Violence, gender and WASH: spurring action on a complex, under-documented and sensitive topic. *Environ. Urban.* **27**, 105–116 (2015).

[24] Remigios, M. V. Women–water–sanitation: the case of Rimuka high-density suburb in Kadoma, Zimbabwe. *Agenda* **25**, 113–121 (2011).

[25] *Female-Friendly Public and Community Toilets: a Guide for Planners and Decision Makers* (UNICEF, WaterAid, and Water and Sanitation for the Urban Poor (WSUP), 2018).

[26] Pouramin, P., Nagabhatla, N. & Miletto, M. A systematic review of water and gender interlinkages: assessing the intersection with health. *Front. Water*10.3389/frwa.2020.00006 (2020).

[27] Khanna, T. & Das, M. Why gender matters in the solution towards safe sanitation? Reflections from rural India. *Glob. Public Health* **11**, 1185–1201 (2016). 26278418 10.1080/17441692.2015.1062905

[28] Kulkarni, S., O’Reilly, K. & Bhat, S. No relief: lived experiences of inadequate sanitation access of poor urban women in India. *Gend. Dev.* **25**, 167–183 (2017).

[29] Borg, S. A. et al. The association between menstrual hygiene, workplace sanitation practices and self-reported urogenital symptoms in a cross-sectional survey of women working in Mukono District, Uganda. *PLoS ONE* **18**, e0288942 (2023). 37471386 10.1371/journal.pone.0288942 PMC10358934

[30] Hirve, S. et al. Psychosocial stress associated with sanitation practices: experiences of women in a rural community in India. *J. Water Sanit. Hyg. Dev.* **5**, 115–126 (2015).

[31] Winter, S., Dreibelbis, R. & Barchi, F. Women’s sanitation practices in informal settlements: a multi-level analysis of factors influencing utilisation in Nairobi, Kenya. *Glob. Public Health* **14**, 663–674 (2019). 30311548 10.1080/17441692.2018.1534256

[32] Faisal, I. M. & Kabir, M. R. An analysis of gender–water nexus in rural Bangladesh. *J. Dev. Soc.* **21**, 175–194 (2005).

[33] Srinivasan R. Lack of toilets and violence against Indian women: empirical evidence and policy implications. *SSRN* https://ssrn.com/abstract=2612052 (2015).

[34] Kwiringira, J., Atekyereza, P., Niwagaba, C. & Günther, I. Descending the sanitation ladder in urban Uganda: evidence from Kampala slums. *BMC Public Health* **14**, 624 (2014). 24948084 10.1186/1471-2458-14-624 PMC4071028

[35] Kwiringira, J., Atekyereza, P., Niwagaba, C. & Günther, I. Gender variations in access, choice to use and cleaning of shared latrines; experiences from Kampala slums, Uganda. *BMC Public Health* **14**, 1180 (2014). 25407788 10.1186/1471-2458-14-1180 PMC4247598

[36] O’Reilly, K. Combining sanitation and women’s participation in water supply: an example from Rajasthan. *Dev. Pract.* **20**, 45–56 (2010).

[37] Sinharoy, S. S., Conrad, A., Patrick, M., McManus, S. & Caruso, B. A. Protocol for development and validation of instruments to measure women’s empowerment in urban sanitation across countries in South Asia and sub-Saharan Africa: the Agency, Resources and Institutional Structures for Sanitation-related Empowerment (ARISE). *BMJ Open* **12**, e053104 (2022). 35177447 10.1136/bmjopen-2021-053104 PMC8860033

[38] Van Eerdewijk, A. et al. *White Paper: a Conceptual Model of Women and Girls’ Empowerment* (Royal Tropical Institute (KIT), 2017).

[39] Sinharoy, S. S., McManus, S., Conrad, A., Patrick, M. & Caruso, B. A. The Agency, Resources, and Institutional Structures for Sanitation-Related Empowerment (ARISE) scales: development and validation of measures of women’s empowerment in urban sanitation for low- and middle-income countries. *World Dev.* **164**, 106183 (2023). 37013085 10.1016/j.worlddev.2023.106183 PMC9918868

[40] Sinharoy, S. S. et al. The Agency, Resources, and Institutional Structures for Sanitation-Related Empowerment (ARISE) Scales: psychometric evaluation across Asia and Africa. Preprint at *Research Square*10.21203/rs.3.rs-4571408/v1 (2024).

[41] Sinharoy, S. S. et al. *The Agency, Resources, and Institutional Structures for Sanitation-Related Empowerment (ARISE) Scale: User Guide* (Evidence and Data on Gender and the Environment (EDGE), 2023); edge.sph.emory.edu

[42] *Global Surface Summary of the Day*—*GSOD* (NOAA National Centers of Environmental Information, 2024).

[43] *Kampala City Sanitation Profile* (Kampala Capital City Authority, 2017); https://www.kcca.go.ug/media/docs/CITYWIDE%20SANITATION%20PROFILE%201-converted.pdf

[44] *Progress on Household Drinking Water, Sanitation and Hygiene 2000–2020: Five Years into the SDGs* (World Health Organization (WHO) and the United Nations Children’s Fund (UNICEF), 2021).

[45] Tumwebaze, I. K., Orach, C. G., Niwagaba, C., Luthi, C. & Mosler, H. J. Sanitation facilities in Kampala slums, Uganda: users’ satisfaction and determinant factors. *Int. J. Environ. Health Res.* **23**, 191–204 (2013). 22873693 10.1080/09603123.2012.713095

[46] *Sanitation Situation Assessment: Tiruchirappalli* (Tamil Nadu Urban Sanitation Support Programme (TNUSSP), 2018); https://www.susana.org/_resources/documents/default/3-3784-226-1615554748.pdf

[47] Kuang, J. et al. Women are more likely to expect social sanctions for open defecation: evidence from Tamil Nadu India. *PLoS ONE* **15**, e0240477 (2020). 33048969 10.1371/journal.pone.0240477 PMC7553302

[48] Babbar, K., Das, U., Ashraf, S., Shpenev, A. & Bicchieri, C. Unlocking the role of social norms: how they shape women’s public toilet usage in India. *Am. J. Trop. Med. Hyg.* **109**, 1177–1186 (2023). 37917999 10.4269/ajtmh.23-0220 PMC10622457

[49] *Gender and Social Inclusion Across the Sanitation Chain in Tamil Nadu—Assessment and Strategy* (Tamil Nadu Urban Sanitation Support Programme (TNUSSP), 2019); https://tnussp.co.in/wp-content/uploads/2020/10/Gender-report-Oct-14-2019.pdf

[50] Smits, J. & Steendijk, R. The international wealth index (IWI). *Soc. Indic. Res.* **122**, 65–85 (2014).

[51] Sinclair, E. et al. Sanitation-related withholding and suppression among women in urban Uganda and India. *figshare*10.6084/m9.figshare.28563911 (2025). 10.1038/s44221-025-00452-5 PMC12279531 40704047

