# Supplementary material for: Sanitation-related withholding and suppression among women in urban Uganda and India
Source: Nat Water. 2025 Jun 17;3(7):782–92. doi: 10.1038/s44221-025-00452-5 (PMC12279531; doi:10.1038/s44221-025-00452-5)
Supplement: Supplementary file 1 — Supplementary Tables 1–12, Figs. 1–8 and Text 1. [file 44221_2025_452_MOESM1_ESM.pdf]

nature water

Supplementary information https://doi.org/10.1038/s44221-025-00452-5

Sanitation-related withholding and suppression among women in urban Uganda and India

In the format provided by the authors and unedited

Sanitation-related withholding and suppression among women in urban Uganda and India:

Supplementary Information

Elaina Sinclair[1], Anke Hüls[1,2], Madeleine Patrick[3], Srishty Arun[4], Vinod Ramanarayanan [4], Sheela S. Sinharoy[2,3], Bethany A. Caruso[2,3]

1. Department of Epidemiology, Rollins School of Public Health, Emory University, 1518 Clifton Rd NE, Atlanta, GA , 30322, USA

2. Gangarosa Department of Environmental Health, Rollins School of Public Health, Emory University, 1518 Clifton Rd NE, Atlanta, GA, 30322, USA

3. Hubert Department of Global Health, Rollins School of Public Health, Emory University, 1518 Clifton Rd NE, Atlanta, GA, 30322, USA

4. Civic Fulcrum, Chennai, India

Correspondence to: Bethany A. Caruso, Hubery Department of Global Health, Rollins School of Public Health, Emory University, 1518 Clifton Rd NE, Atlanta, GA, 30322, USA.

Email: bcaruso@emory.edu

Table of Contents

Supplemental Tables

Supplemental Tables 1a-c: ARISE Scales and their component factors and survey items..................3-5

1a. Privacy Scale...3
1b. Safety and Security Scale...4
1c. Health Scale...5

Supplemental Table 2. Items included in the asset indices for each country according to the WHO International Wealth Index...6

Supplemental Table 3. Women's responses to individual withholding survey items, by setting.............7

Supplemental Table 4. Women's responses to individual suppression survey items, by setting............8

Supplemental Table 5. Parameter estimates and 95% confidence intervals for the withholding Privacy scale models for Kampala and Tiruchirappalli...9

Supplemental Table 6. Parameter estimates and 95% confidence intervals for the withholding Safety and Security scale models for Kampala and Tiruchirappalli...10

Supplemental Table 7. Parameter estimates and 95% confidence intervals for the withholding Safety and Security factor models for Kampala and Tiruchirappalli...11

Supplemental Table 8. Parameter estimates and 95% confidence intervals for the withholding Health factor models for Kampala and Tiruchirappalli..12

Supplemental Table 9. Parameter estimates and 95% confidence intervals for the suppression Privacy scale models for Kampala and Tiruchirappalli...13

Supplemental Table 10. Parameter estimates and 95% confidence intervals for the suppression Safety and Security scale models for Kampala and Tiruchirappalli...14

Supplemental Table 11. Parameter estimates and 95% confidence intervals for the suppression Safety and Security factor models for Kampala and Tiruchirappalli...15

Supplemental Table 12. Parameter estimates and 95% confidence intervals for the suppression Health factor models for Kampala and Tiruchirappalli...16

Supplemental Figures

Supplemental Figure 1: Distribution of mean withholding frequency per woman among women from both Tiruchirappalli and Kampala. 17

Supplemental Figure 2: Distribution of dichotomized withholding variable among women from both Tiruchirappalli and Kampala. 18

Supplemental Figure 3: Distribution of mean suppression frequency among women from both Tiruchirappalli and Kampala 19

Supplemental Figure 4. Privacy scale DAG used to inform covariate selection 20

Supplemental Figure 5. Safety and Security scale DAG used to inform covariate selection 21

Supplemental Figure 6. Health Factors DAG used to inform covariate selection 22

Supplemental Figure 7. Correlations between Safety and Security Scale and Factors 23

Supplemental Figure 8. Correlations between Health Scale and Factors 24

Supplemental Text

Supplemental Text 1. Withholding and Suppression Models 25-26

<u>Supplemental Tables 1a-c.</u> *ARISE Scales and their component factors and survey items. All survey items had the response options of "Never", "Sometimes", "Often" or "Always", coded 0, 1, 2 and 3, respectively.*

1a. Privacy Scale

Factor	Survey Items For Privacy Scale	
Factor 1: Privacy for sanitation	P01	Worried that someone would see me urinating or defecating
	P02	Had to use a sanitation location that was not private enough for me when I was at home
	P03	Had to use a sanitation location that was not private enough for me when I was away from home
	P04	While at home, had to stop urinating or defecating because someone came near me and I no longer had privacy
	P05	While away from home, I had to stop urinating or defecating because someone came near me and I no longer had privacy

1b. Safety and Security Scale

Factors	Survey Items For Safety and Security Scale	
Factor 1: Perceptions of women's risk of harm when going for sanitation	S01	Women in my community face the risk of being physically harmed by men or boys when going to sanitation locations
	S08	Women in my community face the risk of sexual assault when going to sanitation locations
	S10	Women in my community face the risk of someone making sexual comments or saying obscene things to them when they go to sanitation locations
Factor 2: Perceptions of women's risk of harm when going to sanitation-related meetings	S11	Women in my community face the risk of someone making sexual comments or saying obscene things to them when they go to a sanitation-related meeting
	S13	Women in my community face the risk of experiencing harassment, such as being called by rude names, yelling, or shaming, if they go to a sanitation-related meeting
	S14	Women in my community face the risk of experiencing harassment, such as being called by rude names, yelling, or shaming, if they speak up in a sanitation-related meeting
Factor 3: Perceptions of women's risk of domestic violence related to sanitation	S04	Women in my community face the risk of being hit by their husbands or other family members if they go for sanitation without telling someone or stay out too long
	S05	Women in my community face the risk of being hit by their husbands or other family members if they fail to complete sanitation-related chores
	S06	Women in my community face the risk of being hit by their husbands or other family members if they argue with the head of their households/another family member about sanitation issues
	S07	Women in my community face the risk of being hit by their husbands or other family members if they argue with neighbors or other people in the community about sanitation issues
Factor 4: Perceptions of own risk of harm when going for sanitation	S24	Feared I would be physically harmed by someone when I went to a sanitation location when away from home
	S29	Feared someone would make sexual comments or say obscene things to me when I went to a sanitation location when away from home
	S31	Feared someone would expose himself or spy/peep on me when I went to a sanitation location when away from home
	S33	Felt unsafe when using a sanitation location outside the home that was not for women only
Factor 5: Perceptions of general personal safety related to sanitation	S21	Felt unsafe in the place where I typically go for sanitation during the day
	S22	Felt unsafe in a place where I have gone for sanitation when away from home
	S23	Felt unsafe in the place where I typically go for sanitation at night

1c. Health Scale

Health Factors	Survey Items For Health Scale	
Factor 1: Sanitation-related illness	H01	Used a sanitation location that I believed might make me ill
	H02	Got sick as a result of using my sanitation location
	H03	Got sick as a result of cleaning my sanitation location
*Factor 2: Illness due to suppression and withholding	H04	Became ill because I had to suppress the urge to urinate or defecate
	H05	Withholding water to avoid urination made me feel unwell
	H06	Withholding food to avoid defecation made me feel unwell
Factor 3: Fear of injury	H08	Feared being harassed or injured by men, boys, or other people when accessing my sanitation location
	H09	Feared being injured by animals or insects when accessing my sanitation location
	H10	Feared being injured because of the physical conditions - such as slippery conditions, rocks or thorns, uneven pathways, obstacles, sharp doors, or floors, etc. - when accessing my sanitation location
Factor 4: Sanitation-related anxiety, embarrassment, and shame	H12	Felt anxiety, stress, or tension when I needed to access a sanitation location during the day when at home
	H15	Experienced embarrassment or shame when accessing a sanitation location during the day
	H16	Experienced embarrassment or shame when accessing a sanitation location at night
Factor 5: Sanitation-related stress and fear	H17	Been too afraid to use a sanitation location because it is dark
	H18	Felt stress or frustration related to the sanitation conditions in my community
	H19	Felt stress or frustration related to the sanitation conditions in my household
	H20	Feared for the safety of women or children going to sanitation locations

*Health Factor 2 was not used due to the circuitous relationship it introduced between exposure and outcome.

<u>Supplemental Table 2.</u> *Items included in the asset indices for each country according to the WHO International Wealth Index.*

Does your household have (a/an)….	
**Uganda**	**India**
Electricity	Electricity
Radio	Radio or transmitter
Black and white television	Television
Color television	Pressure cooker
Non-mobile television	Sewing machine
Computer	Computer
Refrigerator	Refrigerator
Electric fan	Internet
Table	Table
Chair	Chair
Sofa set	Mattress
Bed	Cot or bed
Cupboard	An air conditioner or fan
Clock	Washing machine
Watch	Water pump
Mobile Phone	Mobile phone
Cassette/CD/DVD Player	Land line phone
Bicycle	Bicycle
Animal-drawn cart	Animal-drawn cart
Motorcycle or scooter	Motorcycle or scooter
Car/truck	Car
Boat with a motor	Autorickshaw
Boat without a motor	Lorry/truck
Agricultural land	Agricultural land
Farm animals (local cattle, exotic/cross-breed cattle, horses, donkeys, mules, goats, sheep, chickens, other poultry, pigs)	Farm animals (local cattle, exotic/cross-breed cattle, horses, donkeys, mules, goats, sheep, chickens, other poultry, pigs)
	Thresher
	Tractor

Supplemental Table 3. *Women's responses to individual withholding survey items, by setting.*

Withholding Survey Item	Kampala (n = 697) Frequency (n, %)				Tiruchirappalli (n = 611) Frequency (n, %)			
	Never	Some-times	Often	Always	Never	Some-times	Often	Always
In the past 30 days, I withheld water to avoid the urge to urinate when I knew that I would be away from home.	465 (66.7%)	148 (21.2%)	46 (6.6%)	38 (5.5%)	533 (87.2%)	56 (9.2%)	17 (2.8%)	5 (0.8%)
In the past 30 days, I withheld food to avoid the urge to defecate when I knew that I would be away from home.	564 (80.9%)	76 (10.9%)	40 (5.7%)	17 (2.4%)	547 (89.5%)	45 (7.4%)	10 (1.6%)	9 (1.5%)
In the past 30 days, I withheld water to avoid the urge to urinate when at home during the day or at night.	557 (79.9%)	79 (11.3%)	40 (5.7%)	21 (3.0%)	574 (93.9%)	24 (3.9%)	10 (1.6%)	3 (0.5%)
In the past 30 days, I withheld food to avoid the urge to defecate when at home either during the day or at night.	617 (88.5%)	47 (6.7%)	23 (3.3%)	10 (1.4%)	588 (96.2%)	18 (2.9%)	5 (0.7%)	1 (0.2%)

Supplemental Table 4. *Women's responses to individual suppression survey items, by setting.*

Suppression Survey Item	Kampala (n = 440) Frequency (n, %)				Tiruchirappalli (n = 350) Frequency (n, %)			
	Never	Some-times	Often	Always	Never	Some-times	Often	Always
In the past 30 days, I had to suppress the urge to urinate or defecate during the daytime when at home.	351 (79.8%)	64 (14.5%)	19 (4.3%)	6 (1.4%)	319 (91.1%)	22 (6.3%)	7 (2.0%)	2 (0.6%)
In the past 30 days, I had to suppress the urge to urinate or defecate at night when at home.	55 (12.5%)	76 (17.3%)	75 (17.0%)	234 (53.2%)	25 (7.1%)	22 (6.3%)	78 (22.3%)	225 (64.2%)
In the past 30 days, I had to suppress the urge to urinate or defecate when I am away from home.	264 (60.0%)	127 (28.9%)	38 (8.6%)	11 (2.5%)	301 (86.0%)	40 (11.4%)	6 (1.7%)	3 (0.9%)

Supplemental Table 5. *Parameter estimates and 95% confidence intervals for the withholding Privacy scale models for Kampala and Tiruchirappalli.*

**Withholding - Privacy Scale**						
Fixed Effects						
	**Kampala, Uganda**			**Tiruchirappalli, India**		
**Parameter**	**Parameter Estimate**	**95% Min**	**95% Max**	**Parameter Estimate**	**95% Min**	**95% Max**
**Intercept**	0.04	0.01	0.23	0.04	0.00	0.73
**Privacy Scale**	5.06	3.38	7.59	2.38	1.22	4.63
**Age**	0.99	0.97	1.01	1.01	0.99	1.03
**Wealth Index**	0.93	0.81	1.07	1.16	0.98	1.37
**Number of Household Members**	1.07	0.97	1.17	0.84	0.71	0.99
**Number of Household Children**	0.74	0.47	1.17	0.88	0.51	1.52
**Hours Away From Home**	1.02	0.98	1.06	1.04	0.98	1.11
**Physical Health (Ref = Excellent)**						
Very Good	2.28	1.06	4.91	1.02	0.40	2.59
Good	1.30	0.63	2.67	0.71	0.31	1.61
Fair	1.39	0.62	3.16	0.98	0.43	2.22
Poor	1.75	0.71	4.32	1.11	0.34	3.64
**Marital Status (Ref = Married)**						
Never Married	1.35	0.81	2.26	1.68	0.77	3.63
Separated/Divorced/Widowed	1.69	1.13	2.52	0.67	0.31	1.46
**Facility Type (Ref = Improved)**						
Unimproved/Open Defecation	NA	NA	NA	5.67	0.49	66.09
**Share Facility (Ref = General Public)**						
Known Households	2.34	1.24	4.45	0.50	0.16	1.55
Privately Owned/Not Shared	3.12	1.40	6.96	0.75	0.34	1.68
**Private Location**	1.51	0.89	2.54	0.92	0.56	1.50
**Could Be Seen Using**	0.61	0.36	1.02	0.47	0.13	1.78
**Men Also Use the Facility**	1.71	0.61	4.76	1.26	0.60	2.64
**Lockable Facility**	0.81	0.50	1.31	0.60	0.23	1.57
**Lighting Inside Facility**	1.13	0.74	1.73	8.94	0.81	98.50
**Lighting Outside/On the Way**	1.05	0.62	1.76	0.48	0.15	1.53
**Physically Challenging to Access**	0.90	0.52	1.54	1.61	0.83	3.15
**Facility Malfunction**	1.10	0.70	1.74	1.63	0.67	3.93
**Collect Water for Household Sanitation Needs**	0.34	0.18	0.66	1.09	0.65	1.81

Supplemental Table 6. *Parameter estimates and 95% confidence intervals for the withholding Safety and Security scale models for Kampala and Tiruchirappalli.*

**Withholding – Safety and Security Scale**						
Fixed Effects						
	**Kampala, Uganda**			**Tiruchirappalli, India**		
**Parameter**	**Parameter Estimate**	**95% Min**	**95% Max**	**Parameter Estimate**	**95% Min**	**95% Max**
**Intercept**	0.03	0.00	0.17	0.02	0.00	0.31
**Safety and Security Scale**	4.28	2.99	6.13	3.26	1.90	5.59
**Age**	0.99	0.97	1.01	1.01	0.99	1.03
**Wealth Index**	0.92	0.80	1.06	1.15	0.97	1.37
**Number of Household Members**	1.06	0.97	1.15	0.86	0.72	1.01
**Number of Household Children**	0.68	0.43	1.07	0.78	0.45	1.35
**Hours Away From Home**	1.02	0.98	1.06	1.03	0.97	1.10
**Physical Health (Ref = Excellent)**						
Very Good	1.97	0.92	4.21	1.13	0.44	2.90
Good	1.21	0.59	2.46	0.71	0.31	1.63
Fair	1.32	0.59	2.96	0.87	0.38	2.00
Poor	1.51	0.62	3.70	1.05	0.32	3.46
**Marital Status (Ref = Married)**						
Never Married	1.27	0.77	2.11	1.65	0.75	3.63
Separated/Divorced/Widowed	1.61	1.08	2.40	0.68	0.31	1.49
**Facility Type (Ref = Improved)**						
Unimproved/Open Defecation	NA	NA	NA	2.47	0.25	24.66
**Share Facility (Ref = General Public)**						
Known Households	2.06	1.11	3.82	0.45	0.14	1.40
Privately Owned/Not Shared	3.00	1.36	6.59	0.74	0.33	1.69
**Private Location**	1.44	0.85	2.44	1.09	0.65	1.82
**Could Be Seen Using**	0.68	0.41	1.12	0.43	0.11	1.66
**Men Also Use the Facility**	1.68	0.59	4.79	1.24	0.58	2.68
**Lockable Facility**	0.77	0.48	1.25	0.55	0.21	1.46
**Lighting Inside Facility**	0.98	0.65	1.50	5.18	0.57	47.13
**Lighting Outside/On the Way**	1.00	0.60	1.69	0.53	0.16	1.71
**Physically Challenging to Access**	0.97	0.57	1.65	1.33	0.66	2.67
**Facility Malfunction**	1.16	0.74	1.82	1.69	0.70	4.06
**Collect Water for Household Sanitation Needs**	0.40	0.20	0.77	1.04	0.62	1.76

<u>Supplemental Table 7.</u> *Parameter estimates and 95% confidence intervals for the withholding Safety and Security factor models for Kampala and Tiruchirappalli.*

Withholding - Safety and Security Factors						
Fixed Effects						
Parameter	Kampala, Uganda (n = 697)			Tiruchirappalli, India (n = 611)		
	Parameter Estimate	95% Min	95% Max	Parameter Estimate	95% Min	95% Max
**Intercept**	0.03	0.00	0.20	0.04	0.00	0.62
**Safety and Security Factor 1**: Perceptions of women's general risk of harm when going for sanitation	0.89	0.61	1.29	1.29	0.78	2.14
**Safety and Security Factor 2**: Perceptions of women's risk of harm when going to sanitation-related meetings	1.33	0.90	1.95	1.27	0.76	2.14
**Safety and Security Factor 3**: Perceptions of women's risk of domestic violence related to sanitation	1.41	1.04	1.90	1.13	0.75	1.71
**Safety and Security Factor 4**: Perceptions of one's own risk of harm when going for sanitation	3.01	2.11	4.29	2.44	1.58	3.77
**Safety and Security Factor 5**: Perceptions of general personal safety related to sanitation	0.90	0.67	1.19	0.68	0.43	1.10
**Age**	0.99	0.98	1.01	1.01	0.99	1.03
**Wealth Index**	0.90	0.78	1.03	1.11	0.93	1.33
**Number of Household Members**	1.04	0.95	1.14	0.84	0.71	1.00
**Number of Household Children**	0.72	0.45	1.14	0.76	0.43	1.33
**Hours Away From Home**	1.04	0.99	1.08	1.05	0.98	1.12
**Physical Health (Ref = Excellent)**						
Very Good	2.17	0.99	4.78	0.94	0.36	2.43
Good	1.42	0.67	2.98	0.56	0.24	1.30
Fair	1.47	0.64	3.40	0.73	0.32	1.71
Poor	1.63	0.66	4.06	0.86	0.26	2.88
**Marital Status (Ref = Married)**						
Never Married	1.31	0.78	2.19	1.64	0.73	3.70
Separated/Divorced/Widowed	1.59	1.06	2.39	0.69	0.31	1.54
**Facility Type (Ref = Improved)**						
Unimproved/Open Defecation	NA	NA	NA	1.74	0.17	18.00
**Sharing Status (Ref = General Public)**						
Known Households	2.08	1.10	3.97	0.42	0.13	1.33
Privately Owned/Not Shared	3.02	1.33	6.83	0.67	0.29	1.52
**Private Location**	1.47	0.86	2.51	0.92	0.54	1.55
**Could Be Seen Using**	0.69	0.41	1.14	0.50	0.13	1.92
**Men Also Use the Facility**	1.72	0.59	4.99	1.30	0.60	2.79
**Lockable Facility**	0.70	0.43	1.15	0.55	0.20	1.50
**Lighting Inside Facility**	1.00	0.65	1.53	4.77	0.50	45.11
**Lighting Outside/On the Way**	0.96	0.57	1.62	0.54	0.15	1.86
**Physically Challenging to Access**	0.99	0.58	1.71	1.46	0.73	2.95
**Facility Malfunction**	1.21	0.76	1.92	1.64	0.67	4.00
**Collect Water for Household Sanitation Needs**	0.38	0.20	0.75	1.07	0.63	1.81

Supplemental Table 8. *Parameter estimates and 95% confidence intervals for the withholding Health factor models for Kampala and Tiruchirappalli.*

**Withholding - Health Factors**						
Fixed Effects						
**Parameter**	**Kampala, Uganda (n = 697)**			**Tiruchirappalli, India (n = 611)**		
	Parameter Estimate	95% Min	95% Max	Parameter Estimate	95% Min	95% Max
**Intercept**	0.01	0.00	0.10	0.02	0.00	0.46
**Health Factor 1:** Sanitation-related illness	1.70	1.25	2.31	2.52	1.01	6.28
**Health Factor 3**: Fear of injury	1.92	1.23	2.98	0.85	0.30	2.39
**Health Factor 4:** Sanitation-related anxiety, embarrassment, and shame	1.32	0.80	2.20	0.70	0.23	2.17
**Health Factor 5:** Sanitation-related stress and fear	1.44	1.09	1.89	2.93	1.59	5.41
**Age**	0.99	0.97	1.01	1.01	0.99	1.03
**Wealth Index**	0.93	0.81	1.07	1.16	0.97	1.38
**Number of Household Members**	1.06	0.97	1.16	0.84	0.71	1.00
**Number of Household Children**	0.78	0.49	1.24	0.83	0.47	1.45
**Hours Away From Home**	1.02	0.98	1.06	1.04	0.97	1.10
**Physical Health (Ref = Excellent)**						
Very Good	2.31	1.07	4.99	0.96	0.38	2.47
Good	1.29	0.62	2.66	0.57	0.25	1.33
Fair	1.41	0.62	3.21	0.82	0.36	1.89
Poor	1.87	0.77	4.56	0.91	0.28	3.01
**Marital Status (Ref = Married)**						
Never Married	1.21	0.72	2.04	1.74	0.79	3.84
Separated/Divorced/Widowed	1.57	1.05	2.34	0.68	0.31	1.51
**Facility Type (Ref = Improved)**						
Unimproved/Open Defecation	NA	NA	NA	4.17	0.40	43.58
**Sharing Status (Ref = General Public)**						
Known Households	2.16	1.15	4.06	0.58	0.19	1.77
Privately Owned/Not Shared	3.30	1.47	7.41	0.70	0.31	1.59
**Private Location**	1.60	0.94	2.72	0.92	0.54	1.55
**Could Be Seen Using**	0.71	0.43	1.17	0.45	0.12	1.67
**Men Also Use the Facility**	1.54	0.54	4.42	1.33	0.63	2.83
**Lockable Facility**	0.91	0.56	1.50	0.62	0.23	1.70
**Lighting Inside Facility**	1.16	0.76	1.76	5.43	0.59	50.40
**Lighting Outside/On the Way**	1.18	0.69	2.03	0.55	0.16	1.88
**Physically Challenging to Access**	0.85	0.49	1.46	1.21	0.60	2.45
**Facility Malfunction**	1.13	0.71	1.79	1.84	0.75	4.49
**Collect Water for Household Sanitation Needs**	0.42	0.21	0.83	1.10	0.65	1.86

<u>Supplemental Table 9.</u> *Parameter estimates and 95% confidence intervals for the suppression Privacy scale models for Kampala and Tiruchirappalli.*

**Suppression - Privacy Scale**						
Fixed Effects						
	**Kampala, Uganda**			**Tiruchirappalli, India**		
**Parameter**	**Parameter Estimate**	**95% Min**	**95% Max**	**Parameter Estimate**	**95% Min**	**95% Max**
**Intercept**	0.04	0.01	0.23	0.04	0.00	0.73
**Privacy Scale**	5.06	3.38	7.59	2.38	1.22	4.63
**Age**	0.99	0.97	1.01	1.01	0.99	1.03
**Wealth Index**	0.93	0.81	1.07	1.16	0.98	1.37
**Number of Household Members**	1.07	0.97	1.17	0.84	0.71	0.99
**Number of Household Children**	0.74	0.47	1.17	0.88	0.51	1.52
**Hours Away From Home**	1.02	0.98	1.06	1.04	0.98	1.11
**Physical Health (Ref = Excellent)**						
Very Good	2.28	1.06	4.91	1.02	0.40	2.59
Good	1.30	0.63	2.67	0.71	0.31	1.61
Fair	1.39	0.62	3.16	0.98	0.43	2.22
Poor	1.75	0.71	4.32	1.11	0.34	3.64
**Marital Status (Ref = Married)**						
Never Married	1.35	0.81	2.26	1.68	0.77	3.63
Separated/Divorced/Widowed	1.69	1.13	2.52	0.67	0.31	1.46
**Facility Type (Ref = Improved)**						
Unimproved/Open Defecation	NA	NA	NA	5.67	0.49	66.09
**Share Facility (Ref = General Public)**						
Known Households	2.34	1.24	4.45	0.50	0.16	1.55
Privately Owned/Not Shared	3.12	1.40	6.96	0.75	0.34	1.68
**Private Location**	1.51	0.89	2.54	0.92	0.56	1.50
**Could Be Seen Using**	0.61	0.36	1.02	0.47	0.13	1.78
**Men Also Use the Facility**	1.71	0.61	4.76	1.26	0.60	2.64
**Lockable Facility**	0.81	0.50	1.31	0.60	0.23	1.57
**Lighting Inside Facility**	1.13	0.74	1.73	8.94	0.81	98.50
**Lighting Outside/On the Way**	1.05	0.62	1.76	0.48	0.15	1.53
**Physically Challenging to Access**	0.90	0.52	1.54	1.61	0.83	3.15
**Facility Malfunction**	1.10	0.70	1.74	1.63	0.67	3.93
**Collect Water for Household Sanitation Needs**	0.34	0.18	0.66	1.09	0.65	1.81

Supplemental Table 10. *Parameter estimates and 95% confidence intervals for the suppression Safety and Security scale models for Kampala and Tiruchirappalli.*

Suppression – Safety and Security Scale						
Fixed Effects						
	Kampala, Uganda			Tiruchirappalli, India		
**Parameter**	**Parameter Estimate**	**95% Min**	**95% Max**	**Parameter Estimate**	**95% Min**	**95% Max**
**Intercept**	1.86	1.41	2.32	1.75	1.30	2.21
**Safety and Security Scale**	0.02	-0.06	0.11	0.09	0.00	0.18
**Age**	0.00	0.00	0.01	0.00	-0.01	0.01
**Wealth Index**	0.01	-0.03	0.04	0.05	0.02	0.08
**Number of Household Members**	0.02	0.00	0.05	-0.01	-0.03	0.00
**Number of Household Children**	-0.02	-0.14	0.10	-0.03	-0.12	0.07
**Hours Away From Home**	0.00	-0.01	0.01	0.00	-0.02	0.01
**Physical Health (Ref = Excellent)**						
Very Good	0.10	-0.09	0.28	0.03	-0.12	0.18
Good	0.00	-0.17	0.18	-0.03	-0.16	0.09
Fair	0.01	-0.20	0.21	-0.15	-0.29	-0.02
Poor	0.17	-0.06	0.40	-0.02	-0.31	0.27
**Marital Status (Ref = Married)**						
Never Married	0.23	0.11	0.35	-0.04	-0.17	0.10
Separated/Divorced/Widowed	0.16	0.06	0.27	-0.03	-0.29	0.22
**Facility Type (Ref = Improved)**						
Unimproved/Open Defecation	NA	NA	NA	-0.04	-0.37	0.28
**Share Facility (Ref = General Public)**						
Known Households	0.03	-0.12	0.18	0.10	-0.10	0.30
Privately Owned/Not Shared	0.08	-0.13	0.28	0.08	-0.09	0.25
**Private Location**	0.01	-0.12	0.14	0.02	-0.07	0.11
**Could Be Seen Using**	-0.03	-0.15	0.10	0.00	-0.18	0.19
**Men Also Use the Facility**	0.00	-0.24	0.24	0.07	-0.09	0.23
**Lockable Facility**	0.08	-0.04	0.20	0.02	-0.19	0.23
**Lighting Inside Facility**	-0.06	-0.17	0.05	-0.06	-0.30	0.19
**Lighting Outside/On the Way**	0.00	-0.13	0.14	-0.09	-0.33	0.15
**Physically Challenging to Access**	0.01	-0.12	0.14	-0.08	-0.24	0.08
**Facility Malfunction**	0.03	-0.09	0.14	-0.04	-0.20	0.12
**Collect Water for Household Sanitation Needs**	-0.30	-0.46	-0.13	0.02	-0.06	0.11

Supplemental Table 11. *Parameter estimates and 95% confidence intervals for the suppression Safety and Security factor models for Kampala and Tiruchirappalli.*

**Suppression - Safety and Security Factors**						
Fixed Effects						
**Parameter**	**Kampala, Uganda (n = 697)**			**Tiruchirappalli, India (n = 611)**		
	Parameter Estimate	95% Min	95% Max	Parameter Estimate	95% Min	95% Max
**Intercept**	1.91	1.45	2.36	1.84	1.38	2.29
**Safety and Security Factor 1**: Perceptions of women's general risk of harm when going for sanitation	-0.19	-0.29	-0.10	0.00	-0.09	0.09
**Safety and Security Factor 2**: Perceptions of women's risk of harm when going to sanitation-related meetings	0.13	0.03	0.22	0.01	-0.08	0.10
**Safety and Security Factor 3**: Perceptions of women's risk of domestic violence related to sanitation	0.07	-0.01	0.14	0.00	-0.07	0.07
**Safety and Security Factor 4**: Perceptions of one's own risk of harm when going for sanitation	0.04	-0.04	0.13	0.13	0.06	0.21
**Safety and Security Factor 5**: Perceptions of general personal safety related to sanitation	-0.02	-0.09	0.04	-0.06	-0.13	0.01
**Age**	0.00	0.00	0.01	0.00	-0.01	0.01
**Wealth Index**	0.01	-0.02	0.05	0.04	0.01	0.07
**Number of Household Members**	0.03	0.00	0.05	-0.02	-0.03	0.00
**Number of Household Children**	-0.04	-0.16	0.08	-0.02	-0.12	0.07
**Hours Away From Home**	0.00	-0.01	0.01	0.00	-0.02	0.01
**Physical Health (Ref = Excellent)**						
Very Good	0.07	-0.12	0.25	0.02	-0.13	0.17
Good	-0.02	-0.19	0.16	-0.05	-0.18	0.07
Fair	-0.06	-0.26	0.15	-0.16	-0.29	-0.02
Poor	0.13	-0.10	0.36	0.01	-0.28	0.30
**Marital Status (Ref = Married)**						
Never Married	0.22	0.10	0.34	-0.03	-0.17	0.10
Separated/Divorced/Widowed	0.15	0.05	0.26	-0.01	-0.26	0.24
**Facility Type (Ref = Improved)**						
Unimproved/Open Defecation	NA	NA	NA	-0.09	-0.41	0.24
**Sharing Status (Ref = General Public)**						
Known Households	-0.01	-0.16	0.14	0.08	-0.11	0.28
Privately Owned/Not Shared	0.01	-0.20	0.21	0.07	-0.10	0.24
**Private Location**	0.03	-0.10	0.16	0.00	-0.09	0.09
**Could Be Seen Using**	-0.02	-0.14	0.10	0.03	-0.16	0.21
**Men Also Use the Facility**	0.02	-0.22	0.25	0.07	-0.08	0.23
**Lockable Facility**	0.03	-0.09	0.15	0.02	-0.19	0.23
**Lighting Inside Facility**	-0.05	-0.16	0.06	-0.08	-0.32	0.16
**Lighting Outside/On the Way**	0.00	-0.14	0.13	-0.07	-0.31	0.17
**Physically Challenging to Access**	0.03	-0.10	0.16	-0.06	-0.22	0.10
**Facility Malfunction**	0.05	-0.07	0.16	-0.05	-0.21	0.11
**Collect Water for Household Sanitation Needs**	-0.27	-0.43	-0.10	0.03	-0.05	0.12

Supplemental Table 12. *Parameter estimates and 95% confidence intervals for the suppression Health factor models for Kampala and Tiruchirappalli.*

Suppression - Health Factors						
Fixed Effects						
**Parameter**	**Kampala, Uganda (n = 697)**			**Tiruchirappalli, India (n = 611)**		
	Parameter Estimate	95% Min	95% Max	Parameter Estimate	95% Min	95% Max
**Intercept**	1.92	1.45	2.39	1.73	1.21	2.25
**Health Factor 1:** Sanitation-related illness	0.07	-0.01	0.15	-0.02	-0.20	0.16
**Health Factor 3**: Fear of injury	0.07	-0.04	0.18	0.20	-0.01	0.42
**Health Factor 4:** Sanitation-related anxiety, embarrassment, and shame	-0.15	-0.28	-0.02	-0.12	-0.33	0.09
**Health Factor 5:** Sanitation-related stress and fear	0.01	-0.06	0.08	0.04	-0.08	0.15
**Age**	0.00	-0.01	0.01	0.00	-0.01	0.01
**Wealth Index**	0.01	-0.02	0.05	0.05	0.02	0.07
**Number of Household Members**	0.02	0.00	0.05	-0.02	-0.03	0.00
**Number of Household Children**	0.00	-0.13	0.12	-0.01	-0.11	0.08
**Hours Away From Home**	0.00	-0.01	0.01	0.00	-0.02	0.01
**Physical Health (Ref = Excellent)**						
Very Good	0.11	-0.08	0.30	0.02	-0.13	0.17
Good	0.00	-0.17	0.18	-0.03	-0.16	0.10
Fair	0.01	-0.19	0.22	-0.15	-0.28	-0.01
Poor	0.20	-0.03	0.43	-0.02	-0.31	0.27
**Marital Status (Ref = Married)**						
Never Married	0.23	0.11	0.36	-0.05	-0.19	0.08
Separated/Divorced/Widowed	0.16	0.06	0.26	-0.02	-0.28	0.24
**Facility Type (Ref = Improved)**						
Unimproved/Open Defecation	NA	NA	NA	0.03	-0.30	0.36
**Sharing Status (Ref = General Public)**						
Known Households	0.01	-0.14	0.16	0.11	-0.08	0.31
Privately Owned/Not Shared	0.06	-0.14	0.26	0.09	-0.08	0.26
**Private Location**	0.02	-0.11	0.15	0.02	-0.08	0.11
**Could Be Seen Using**	-0.02	-0.15	0.10	-0.01	-0.19	0.18
**Men Also Use the Facility**	-0.02	-0.26	0.22	0.07	-0.09	0.23
**Lockable Facility**	0.07	-0.05	0.19	0.08	-0.14	0.30
**Lighting Inside Facility**	-0.06	-0.17	0.05	-0.06	-0.31	0.18
**Lighting Outside/On the Way**	-0.02	-0.15	0.12	-0.08	-0.33	0.17
**Physically Challenging to Access**	0.02	-0.11	0.16	-0.09	-0.26	0.07
**Facility Malfunction**	0.04	-0.08	0.16	-0.04	-0.20	0.12
**Collect Water for Household Sanitation Needs**	-0.32	-0.49	-0.15	0.02	-0.06	0.11

<u>Supplemental Figure 1:</u> *Distribution of mean withholding frequency per woman among women from both Tiruchirappalli and Kampala. Mean withholding frequency calculated as the mean frequency over all four withholding items.*

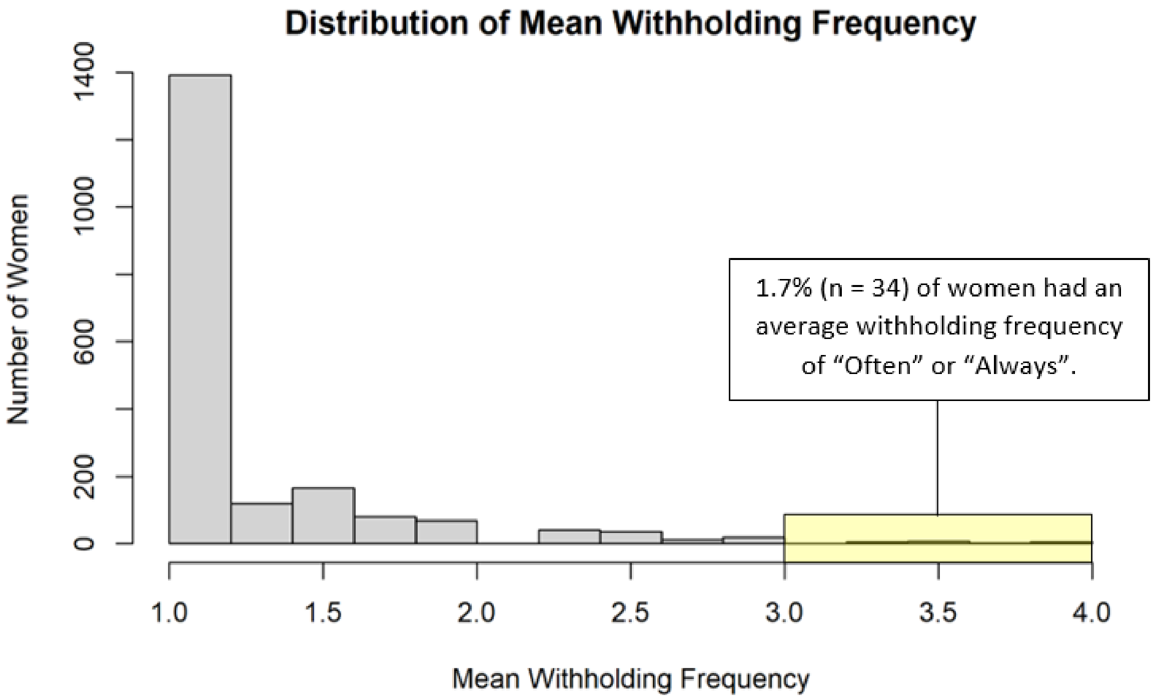

Supplemental Figure 2: *Distribution of dichotomized withholding variable among women from both Tiruchirappalli and Kampala. The withholding variable was dichotomized as zero reported withholding and any non-zero level of withholding reported across the four withholding items.*

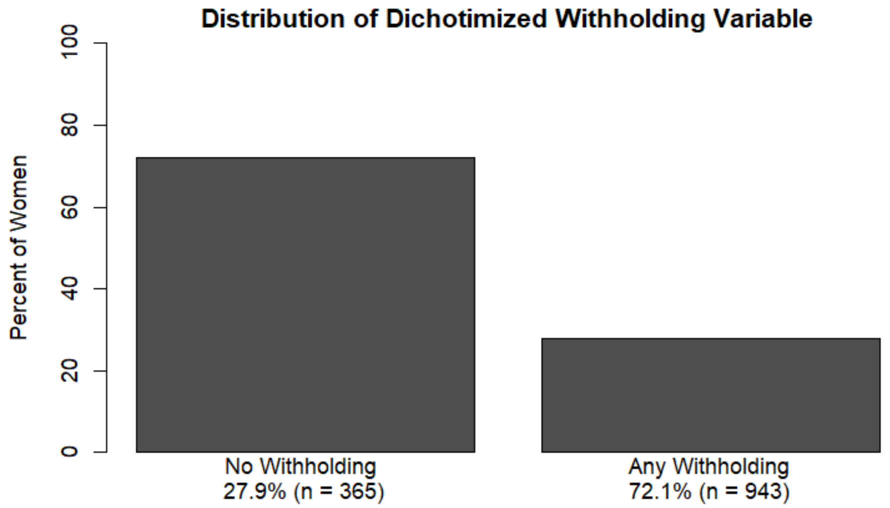

Supplemental Figure 3: *Distribution of mean suppression frequency among women from both Tiruchirappalli and Kampala. Response options were 'Never" – 1, "Sometimes" – 2, "Often" – 3 and "Always" – 4.*

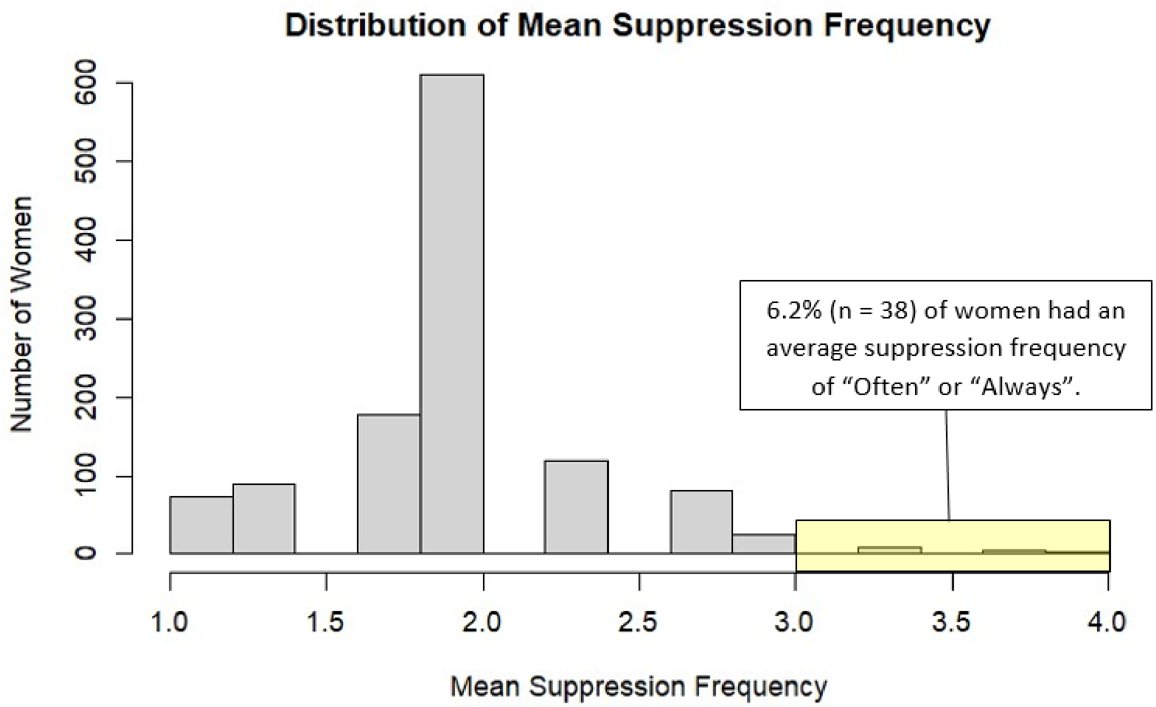

<u>Supplemental Figure 4.</u> *Privacy scale DAG used to inform covariate selection.*

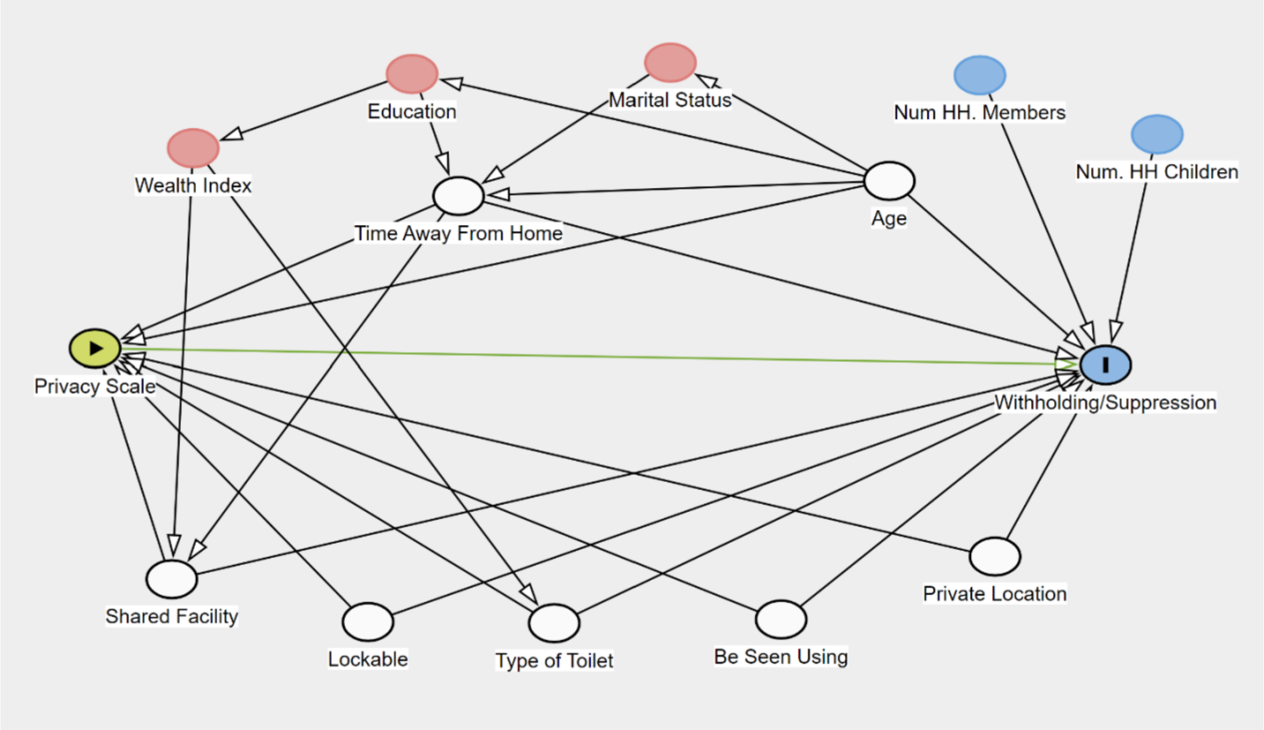

<u>Supplemental Figure 5</u>. *Safety and Security scale DAG used to inform covariate selection.*

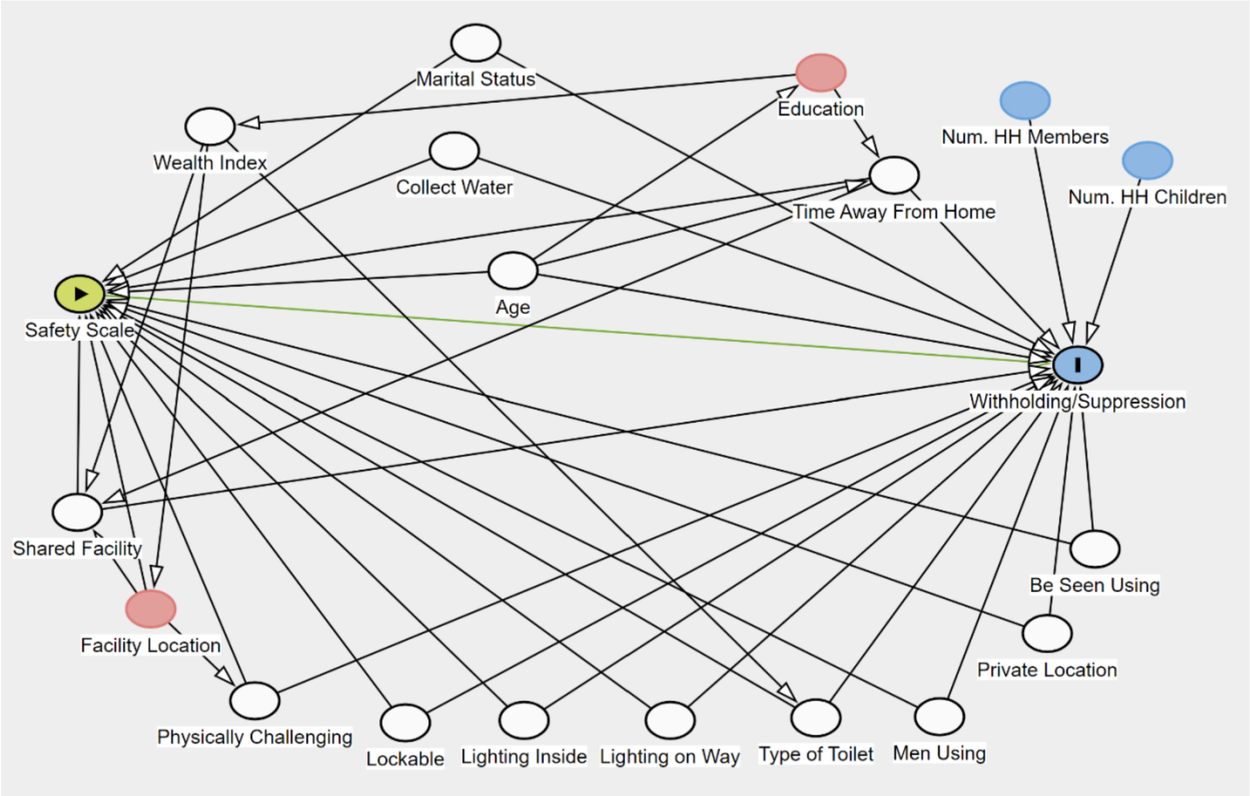

Supplemental Figure 6. *Health Factors DAG used to inform covariate selection.*

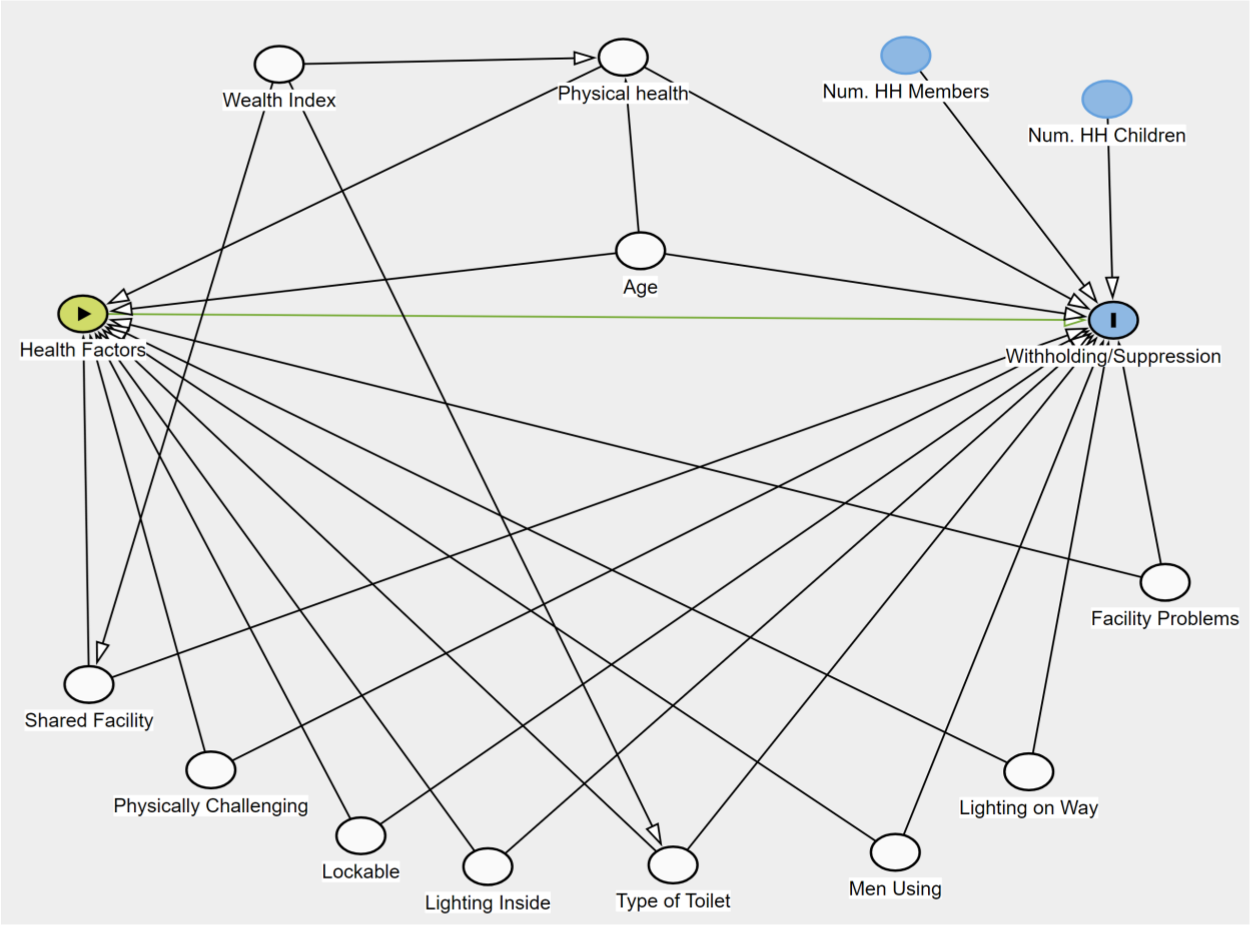

Supplemental Figure 7. Correlations between Safety and Security Scale and Factors

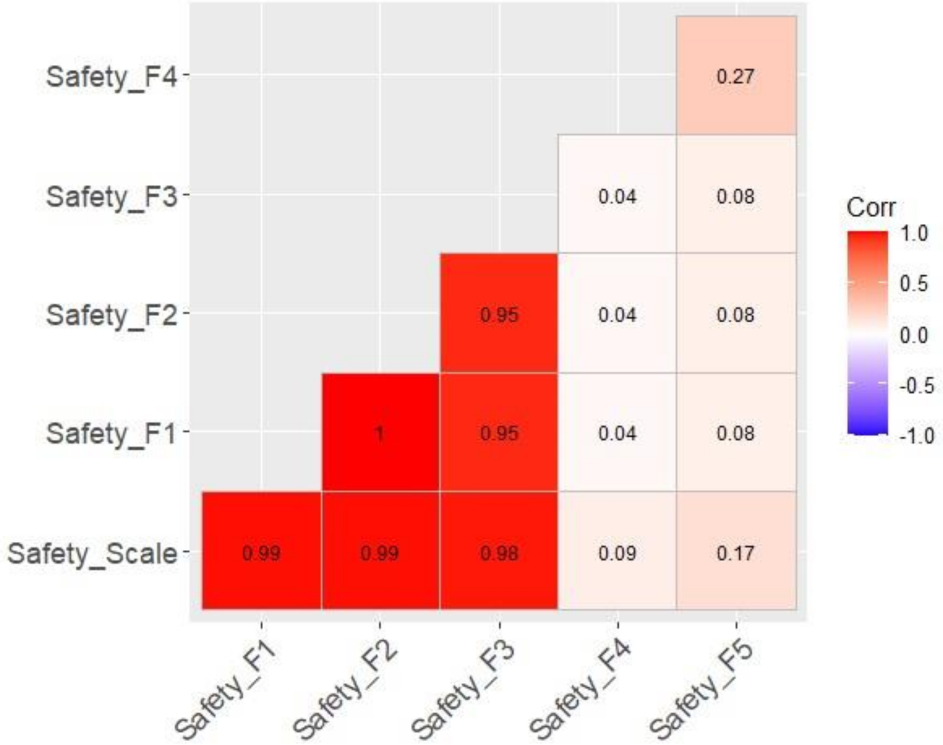

<u>Supplemental Figure 8.</u> Correlations between Health Scale and Factors

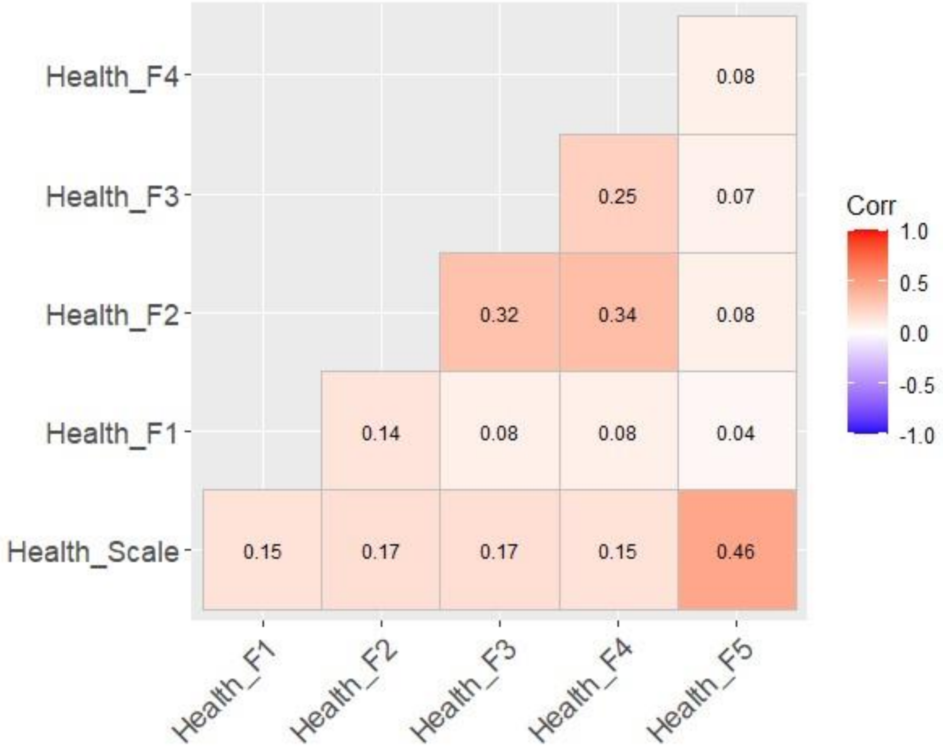

Supplemental Text 1. *Withholding and Suppression Models*

- Withholding and Scale score (Privacy or Safety and Security)
  - $\text{logit}(\textbf{Withholding}) = \alpha + \beta * \text{Scale Score} + \gamma_1 * \text{Age} + \gamma_2 * \text{Wealth Index} + \gamma_3 * \text{Num. Household Members} + \gamma_4 * \text{Num. Household Children} + \gamma_5 * \text{Hours Away From Home} + \gamma_6 * \text{SelfRated Physical Health} + \gamma_7 * \text{Marital Status} + \gamma_8 * \text{Facility Type} + \gamma_9 * \text{Share Facility} + \gamma_{10} * \text{Private Location} + \gamma_{11} * \text{Could Be Seen Using} + \gamma_{12} * \text{Men Use Facility} + \gamma_{13} * \text{Lockable} + \gamma_{14} * \text{Lighting Inside} + \gamma_{15} * \text{Lighting Outside} + \gamma_{16} * \text{Physcially Challenging} + \gamma_{17} * \text{Facility Malfunction} + \gamma_{18} * \text{Collect Water for Household Sanitation}$

- Withholding and Factor score (Safety and Security)
  - $\text{logit}(\textbf{Withholding}) = \alpha + \beta_1 * \text{Safety and Security Factor 1 Score} + \beta_2 * \text{Safety and Security Factor 2 Score} + \beta_3 * \text{Safety and Security Factor 3 Score} + \beta_4 * \text{Safety and Security Factor 4 Score} + \beta_5 * \text{Safety and Security Factor 5 Score} + \gamma_1 * \text{Age} + \gamma_2 * \text{Wealth Index} + \gamma_3 * \text{Num. Household Members} + \gamma_4 * \text{Num. Household Children} + \gamma_5 * \text{Hours Away From Home} + \gamma_6 * \text{SelfRated Physical Health} + \gamma_7 * \text{Marital Status} + \gamma_8 * \text{Facility Type} + \gamma_9 * \text{Share Facility} + \gamma_{10} * \text{Private Location} + \gamma_{11} * \text{Could Be Seen Using} + \gamma_{12} * \text{Men Use Facility} + \gamma_{13} * \text{Lockable} + \gamma_{14} * \text{Lighting Inside} + \gamma_{15} * \text{Lighting Outside} + \gamma_{16} * \text{Physcially Challenging} + \gamma_{17} * \text{Facility Malfunction} + \gamma_{18} * \text{Collect Water for Household Sanitation}$

- Withholding and Factor score (Health)
  - $\text{logit}(\textbf{Withholding}) = \alpha + \beta_1 * \text{Health Factor 1 Score} + \beta_2 * \text{Health Factor 3 Score} + \beta_3 * \text{Health Factor 4 Score} + \beta_4 * \text{HealthFactor 5 Score} + \gamma_1 * \text{Age} + \gamma_2 * \text{Wealth Index} + \gamma_3 * \text{Num. Household Members} + \gamma_4 * \text{Num. Household Children} + \gamma_5 * \text{Hours Away From Home} + \gamma_6 * \text{SelfRated Physical Health} + \gamma_7 * \text{Marital Status} + \gamma_8 * \text{Facility Type} + \gamma_9 * \text{Share Facility} + \gamma_{10} * \text{Private Location} + \gamma_{11} * \text{Could Be Seen Using} + \gamma_{12} * \text{Men Use Facility} + \gamma_{13} * \text{Lockable} + \gamma_{14} * \text{Lighting Inside} + \gamma_{15} * \text{Lighting Outside} + \gamma_{16} * \text{Physcially Challenging} + \gamma_{17} * \text{Facility Malfunction} + \gamma_{18} * \text{Collect Water for Household Sanitation}$

- Suppression and Scale score (Privacy or Safety and Security)
  - $\textbf{Suppression Score} = \alpha + \beta * \text{Scale Score} + \gamma_1 * \text{Age} + \gamma_2 * \text{Wealth Index} + \gamma_3 * \text{Num. Household Members} + \gamma_4 * \text{Num. Household Children} + \gamma_5 * \text{Hours Away From Home} + \gamma_6 * \text{SelfRated Physical Health} + \gamma_7 * \text{Marital Status} + \gamma_8 * \text{Facility Type} + \gamma_9 * \text{Share Facility} + \gamma_{10} * \text{Private Location} + \gamma_{11} * \text{Could Be Seen Using} + \gamma_{12} * \text{Men Use Facility} + \gamma_{13} * \text{Lockable} + \gamma_{14} * \text{Lighting Inside} + \gamma_{15} * \text{Lighting Outside} + \gamma_{16} * \text{Physcially Challenging} + \gamma_{17} * \text{Facility Malfunction} + \gamma_{18} * \text{Collect Water for Household Sanitation}$

- Suppression and Factor score (Safety and Security)
  - $\textbf{Suppression Score} = \alpha + \beta_1 * \text{Safety and Security Factor 1 Score} + \beta_2 * \text{Safety and Security Factor 2 Score} + \beta_3 * \text{Safety and Security Factor 3 Score} + \beta_4 * \text{Safety and Security Factor 4 Score} + \beta_5 * \text{Safety and Security Factor 5 Score} + \gamma_1 * \text{Age} + \gamma_2 * \text{Wealth Index} + \gamma_3 * \text{Num. Household Members} + \gamma_4 * \text{Num. Household Children} + \gamma_5 * \text{Hours Away From Home} + \gamma_6 * \text{SelfRated Physical Health} + \gamma_7 * \text{Marital Status} + \gamma_8 * \text{Facility Type} + \gamma_9 * \text{Share Facility} + \gamma_{10} * \text{Private Location} + \gamma_{11} * \text{Could Be Seen Using} + \gamma_{12} * \text{Men Use Facility} + \gamma_{13} * \text{Lockable} + \gamma_{14} * \text{Lighting Inside} + \gamma_{15} * \text{Lighting Outside} + \gamma_{16} * \text{Physcially Challenging} + \gamma_{17} * \text{Facility Malfunction} + \gamma_{18} * \text{Collect Water for Household Sanitation}$

- Suppression and Factor score (Health)
  - $\textbf{Suppression Score} = \alpha + \beta_1 * \text{Health Factor 1 Score} + \beta_2 * \text{Health Factor 3 Score} + \beta_3 * \text{Health Factor 4 Score} + \beta_4 * \text{HealthFactor 5 Score} + \gamma_1 * \text{Age} + \gamma_2 * \text{Wealth Index} + \gamma_3 * \text{Num. Household Members} + \gamma_4 * \text{Num. Household Children} + \gamma_5 * \text{Hours Away From Home} + \gamma_6 * \text{SelfRated Physical Health} + \gamma_7 * \text{Marital Status} + \gamma_8 * \text{Facility Type} + \gamma_9 * \text{Share Facility} + \gamma_{10} * \text{Private Location} + \gamma_{11} * \text{Could Be Seen Using} + \gamma_{12} * \text{Men Use Facility} + \gamma_{13} * \text{Lockable} + \gamma_{14} * \text{Lighting Inside} + \gamma_{15} * \text{Lighting Outside} + \gamma_{16} * \text{Physcially Challenging} + \gamma_{17} * \text{Facility Malfunction} + \gamma_{18} * \text{Collect Water for Household Sanitation}$
