# Supplementary material for: Sanitation-related withholding and suppression among women in urban Uganda and India
Source: Nat Water. 2025 Jun 17;3(7):782–92. doi: 10.1038/s44221-025-00452-5 (PMC12279531; doi:10.1038/s44221-025-00452-5)
Supplement: Supplementary file 2 — Reporting Summary [file 44221_2025_452_MOESM2_ESM.pdf]

nature portfolio

Corresponding author(s): Bethany Caruso

Last updated by author(s): 05/02/2025

Reporting Summary

Nature Portfolio wishes to improve the reproducibility of the work that we publish. This form provides structure for consistency and transparency in reporting. For further information on Nature Portfolio policies, see our Editorial Policies and the Editorial Policy Checklist.

Statistics

For all statistical analyses, confirm that the following items are present in the figure legend, table legend, main text, or Methods section.

n/a	Confirmed	
☐	☒	The exact sample size (*n*) for each experimental group/condition, given as a discrete number and unit of measurement
☐	☒	A statement on whether measurements were taken from distinct samples or whether the same sample was measured repeatedly
☒	☐	The statistical test(s) used AND whether they are one- or two-sided<br>*Only common tests should be described solely by name; describe more complex techniques in the Methods section.*
☐	☒	A description of all covariates tested
☐	☒	A description of any assumptions or corrections, such as tests of normality and adjustment for multiple comparisons
☐	☒	A full description of the statistical parameters including central tendency (e.g. means) or other basic estimates (e.g. regression coefficient) AND variation (e.g. standard deviation) or associated estimates of uncertainty (e.g. confidence intervals)
☐	☒	For null hypothesis testing, the test statistic (e.g. F, t, r) with confidence intervals, effect sizes, degrees of freedom and P value noted<br>*Give P values as exact values whenever suitable.*
☒	☐	For Bayesian analysis, information on the choice of priors and Markov chain Monte Carlo settings
☒	☐	For hierarchical and complex designs, identification of the appropriate level for tests and full reporting of outcomes
☒	☐	Estimates of effect sizes (e.g. Cohen's *d*, Pearson's *r*), indicating how they were calculated

Our web collection on statistics for biologists contains articles on many of the points above.

Software and code

Policy information about availability of computer code

Data collection	Cross-sectional data were collected in two locations: Kampala, Uganda, and Tiruchirappalli, India in 2019 as part of the Measuring Urban Sanitation and Empowerment (MUSE) project; this is a secondary data analysis of that data.
Data analysis	We used R Studio v4.1.1 to generate descriptive statistics and run regression models.

For manuscripts utilizing custom algorithms or software that are central to the research but not yet described in published literature, software must be made available to editors and reviewers. We strongly encourage code deposition in a community repository (e.g. GitHub). See the Nature Portfolio guidelines for submitting code & software for further information.

Data

Policy information about availability of data

All manuscripts must include a data availability statement. This statement should provide the following information, where applicable:

- Accession codes, unique identifiers, or web links for publicly available datasets
- A description of any restrictions on data availability
- For clinical datasets or third party data, please ensure that the statement adheres to our policy

All data will be made available upon publication in the appendix.

Research involving human participants, their data, or biological material

Policy information about studies with human participants or human data. See also policy information about sex, gender (identity/presentation), and sexual orientation and race, ethnicity and racism.

Reporting on sex and gender	All participants are women; women were intentionally sought for engagement in this research.
Reporting on race, ethnicity, or other socially relevant groupings	Data was collected in Kampala, Uganda, and Tiruchirappalli, India. Data was analyzed by city population, but not by race, ethnicity, or caste.
Population characteristics	For this analysis, populations were described by location, wealth indices, household sizes, and sanitation characteristics.
Recruitment	Participants were recruited for the Measuring Urban Sanitation and Empowerment (MUSE) project. To be eligible for participation in the survey, a woman needed to be 18 or older, speak Luganda (in Uganda) or Tamil (in India) or English, be mentally competent as demonstrated by an ability to understand the survey and consent, and have no speech or hearing impediments (to avoid comprehension difficulties).
Ethics oversight	Study activities were reviewed and approved by Institutional Review Boards at Emory University (USA; IRB 00110271), Azim Premji University (India; reference number 2019/SOD/Faculty/5.1) and Makerere University (Uganda; reference number 2019–038).

Note that full information on the approval of the study protocol must also be provided in the manuscript.

Field-specific reporting

Please select the one below that is the best fit for your research. If you are not sure, read the appropriate sections before making your selection.

☐ Life sciences ☒ Behavioural & social sciences ☐ Ecological, evolutionary & environmental sciences

For a reference copy of the document with all sections, see nature.com/documents/nr-reporting-summary-flat.pdf

Behavioural & social sciences study design

All studies must disclose on these points even when the disclosure is negative.

Study description	Cross-sectional
Research sample	A total of 2,173 women participated in the survey: 1,094 in Kampala and 1,080 in Tiruchirappalli. The analytical samples was 697 and 611 women in Kampala and Tiruchirappalli, respectively.
Sampling strategy	We purposively selected 23 neighborhoods in Tiruchirappalli and 10 parishes in Kampala for survey administration in coordination with implementing partners and local government officials, with a focus on low- to middle-income neighborhoods.
Data collection	Random sampling procedures were used to select households within each neighborhood or parish and targeted an adult woman within each selected household. In each site, 12–14 female enumerators were trained to conduct data collection.
Timing	Data collection lasted 24–30days in each site in December 2019 and January 2020.
Data exclusions	Participant data was excluded from the analytical dataset if there was missing data.
Non-participation	Participants could chose to not answer any question they were unwilling to answer.
Randomization	Participants were not allocated to experimental groups.

Reporting for specific materials, systems and methods

We require information from authors about some types of materials, experimental systems and methods used in many studies. Here, indicate whether each material, system or method listed is relevant to your study. If you are not sure if a list item applies to your research, read the appropriate section before selecting a response.

Materials & experimental systems

n/a	Involved in the study
☒	☐ Antibodies
☒	☐ Eukaryotic cell lines
☒	☐ Palaeontology and archaeology
☒	☐ Animals and other organisms
☒	☐ Clinical data
☒	☐ Dual use research of concern
☒	☐ Plants

Methods

n/a	Involved in the study
☒	☐ ChIP-seq
☒	☐ Flow cytometry
☒	☐ MRI-based neuroimaging

Plants

Seed stocks	*Report on the source of all seed stocks or other plant material used. If applicable, state the seed stock centre and catalogue number. If plant specimens were collected from the field, describe the collection location, date and sampling procedures.*
Novel plant genotypes	*Describe the methods by which all novel plant genotypes were produced. This includes those generated by transgenic approaches, gene editing, chemical/radiation-based mutagenesis and hybridization. For transgenic lines, describe the transformation method, the number of independent lines analyzed and the generation upon which experiments were performed. For gene-edited lines, describe the editor used, the endogenous sequence targeted for editing, the targeting guide RNA sequence (if applicable) and how the editor was applied.*
Authentication	*Describe any authentication procedures for each seed stock used or novel genotype generated. Describe any experiments used to assess the effect of a mutation and, where applicable, how potential secondary effects (e.g. second site T-DNA insertions, mosiacism, off-target gene editing) were examined.*
